# Tension at the gate: sensing mechanical forces at the blood–brain barrier in health and disease

**DOI:** 10.1186/s12974-024-03321-2

**Published:** 2024-12-18

**Authors:** Cathrin E. Hansen, David Hollaus, Alwin Kamermans, Helga E. de Vries

**Affiliations:** 1 Department of Molecular Cell Biology and Immunology, Amsterdam UMC location Vrije Universiteit Amsterdam, De Boelelaan 1117, Amsterdam, The Netherlands; 2 Amsterdam Neuroscience, Amsterdam UMC, Amsterdam, The Netherlands; 3 MS Center Amsterdam, Amsterdam UMC Location VU Medical Center, Amsterdam, The Netherlands

**Keywords:** Blood–brain barrier, Mechanical forces, Wall shear stress, Basement membrane stiffness, Transendothelial migration, TRP, Multiple sclerosis, Alzheimer’s disease, Stroke, Small vessel disease

## Abstract

Microvascular brain endothelial cells tightly limit the entry of blood components and peripheral cells into the brain by forming the blood–brain barrier (BBB). The BBB is regulated by a cascade of mechanical and chemical signals including shear stress and elasticity of the adjacent endothelial basement membrane (BM). During physiological aging, but especially in neurological diseases including multiple sclerosis (MS), stroke, small vessel disease, and Alzheimer’s disease (AD), the BBB is exposed to inflammation, rigidity changes of the BM, and disturbed cerebral blood flow (CBF). These altered forces lead to increased vascular permeability, reduced endothelial reactivity to vasoactive mediators, and promote leukocyte transmigration. Whereas the molecular players involved in leukocyte infiltration have been described in detail, the importance of mechanical signalling throughout this process has only recently been recognized. Here, we review relevant features of mechanical forces acting on the BBB under healthy and pathological conditions, as well as the endothelial mechanosensory elements detecting and responding to altered forces. We demonstrate the underlying complexity by focussing on the family of transient receptor potential (TRP) ion channels. A better understanding of these processes will provide insights into the pathogenesis of several neurological disorders and new potential leads for treatment.

## Background

**Fig. 1:**
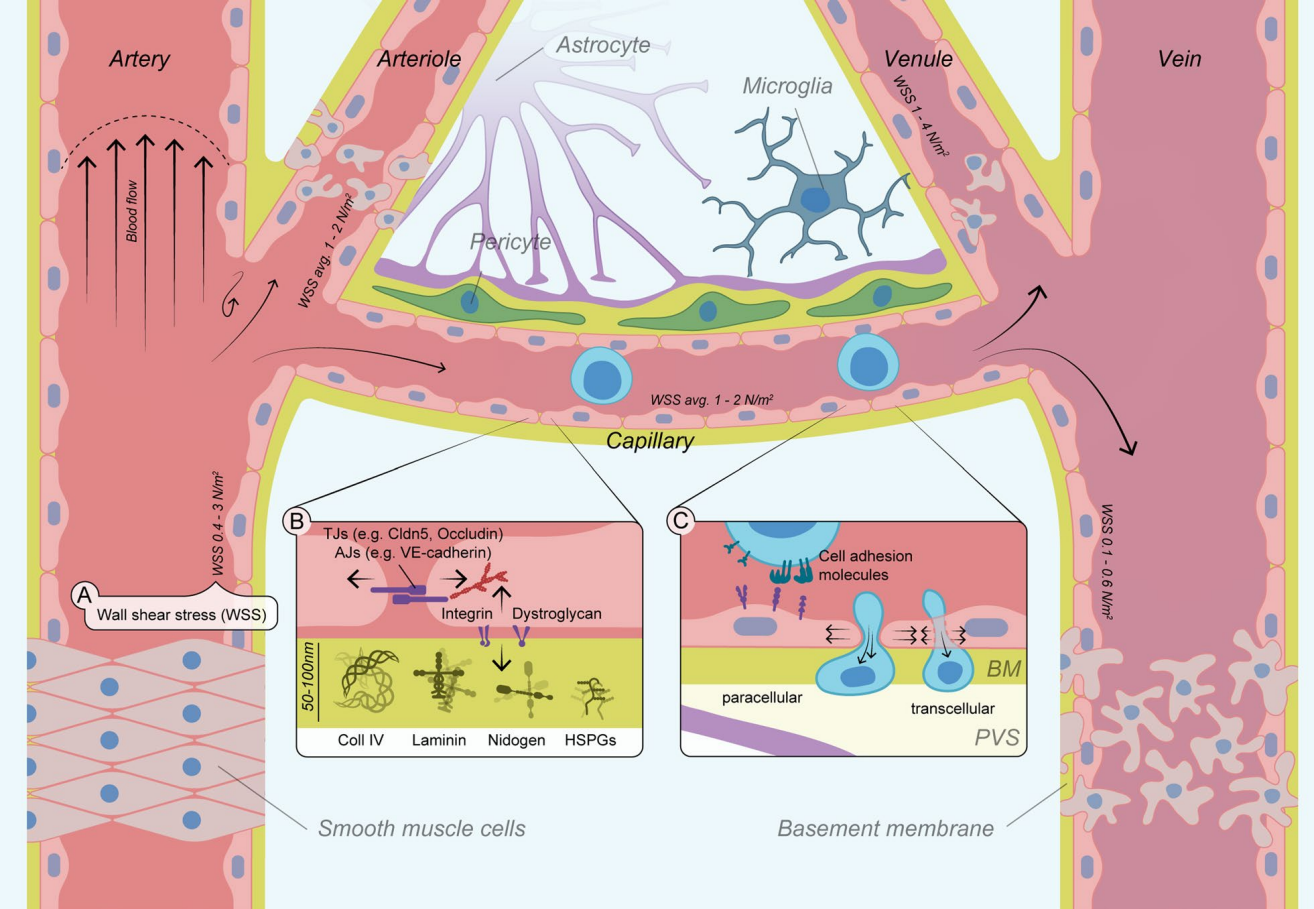
A trio of mechanical forces acting on the brain endothelium. Forces and their directionality are presented by black arrows. **A** Wall shear stress (WSS) acts on the luminal side of the BMECs in the direction of the cerebral blood flow (CBF). **B** Abluminally, BMECs are anchored to the basement membrane (BM) and use integrin-mediated mechanotransduction to respond to the underlying stiffness. Basolateral junction complexes include tight junctions (TJs) and adherens junctions (AJ) creating inter- and intracellular tension. (**C)** Mostly under pathological conditions: Immune cell migration across the BBB is a multi-step cascade with mechanical forces acting on both cellular players, the immune cell, and BMECs. Figure is based on Vanlandewijk et al. [37]. *HSPGs* Heparan sulfate proteoglycans, *Coll IV* Collagen IV, *PVS* perivascular space, *BM* basement membrane

Exchanges of circulating molecules and cells between the bloodstream and central nervous system (CNS) are regulated by the neurovascular unit, a multi-layered structure consisting of perivascular astrocytes, microglia, pericytes, and specialized brain microvascular endothelial cells (BMECs) comprising the blood–brain barrier (BBB) [1–3] (Fig. 1, central part). The BBB is the innermost, monocellular layer of the brain microvasculature forming a tight physical barrier that is characterized by a unique network of adherens junctions (AJs) and tight junctions (TJs) [3, 4], which respond to internal and external mechanical forces of the local microenvironment [5, 6] (Fig. 1B). AJs consist of transmembrane protein complexes, such as vascular endothelial (VE-) cadherin [1, 4], which connect to the intracellular actin cytoskeleton via catenins [4, 7]. TJs are located at the basolateral side of BMECs and include claudin-5, occludin, junctional adhesion molecules (JAMs), and tri-cellular junction proteins such as tricellulin [8–10]. Together, they form a robust paracellular connection and generate intercellular tension, giving rise to the exceptionally low permeability of the BBB [1, 3]. Finally, integrin-based focal adhesions anchor BMECs to the underlying basement membrane (BM), a network of extracellular matrix (ECM) proteins, and an important source of mechanical force (Fig. 1B) [11]. To maintain their integrity, BMECs sense and adapt to changes in forces including cerebral blood flow (CBF), BM elasticity, and cell–cell interactions such as during transendothelial migration (TEM) of peripheral immune cells across the BBB (Fig. 1A–C) [1, 4, 8].

Pathological changes of the BBB are found in many cerebral disorders. In multiple sclerosis (MS), a chronic neuroinflammatory disease, BBB impairment, and immune cell infiltration are disease hallmarks and tightly connected to the formation of demyelinated plaques [12, 13]. Alzheimer’s disease (AD) is characterized by aggregation of amyloid beta (Aβ) and accumulation of hyperphosphorylated tau forming neurofibrillary tangles [14]. These are further accentuated by vascular changes including BBB injury, reduced CBF, and enlarged perivascular spaces [15]. Impaired Aβ clearance is a common feature of AD and cerebral amyloid angiopathy (CAA), whereas in the latter Aβ accumulates particularly around the vasculature, further impairing vascular reactivity and possibly permitting haemorrhages [16]. Reduced vascular function through vessel stiffening and chronic hypertension is commonly found in aging cerebral vasculature and is known to increase the risk for stroke (haemorrhagic and ischemic) and sporadic small vessel disease [17, 18]. Overall, vascular stiffening and disturbed CBF likely result in reduced vascular reactivity, enhanced perivascular aggregate formation, and eventually focal hypoxia, contributing to the (vascular) pathology observed in numerous age-related neurodegenerative disorders [19–28].

Many detrimental vascular changes in cerebral disorders follow and include distinct mechanical components such as alterations of BM composition and wall shear stress (WSS) acting on the microvasculature (Fig. 1A, B). WSS or often referred to as simply shear stress defines the arising force within a moving fluid and between the fluid and vessel wall [29]. Additionally, neuroinflammation facilitates the migration of peripheral immune cells across the BBB, which adds cell–cell interaction forces to the mechanical trio [30, 31] (Fig. 1C). TEM of immune cells into the brain is a hallmark of MS, but also gains increasing attention in AD patients, stroke, and healthy elderly [32, 33]. This migration cascade governs many biomechanical and biochemical interactions from start to finish. Whereas biochemical signalling has been described and reviewed in detail [1, 3, 8, 34], the importance of mechanical stress has only recently been realised.

It becomes apparent that changes in mechanical forces largely influence vascular function and dysfunction and might be early drivers of neuroinflammatory and neurodegenerative diseases. In this review, we briefly summarize our current knowledge of the two main mechanical stressors, WSS and BM stiffness, acting on the BBB and their pathological changes. Then, we highlight the forces measured during TEM of immune cells across the BBB and BBB-specific migration behaviour. Finally, we focus on mechanosensory/sensitive elements of BMECs and spotlight members of the transient receptor potential (TRP) ion channel family to showcase the intricacies of mechanical signalling [35, 36].

### Mechanical forces at the BBB

Cerebral vessels are subjected to a range of mechanical stressors (Table 1), which play a key role in BBB maintenance, activation, and function [38, 39]. Changes in mechanical stimuli are sensed by specialized mechanosensors and translated into biochemical signalling cascades [40], culminating in cytoskeletal reorganization, gene regulation, and epigenetic chromatin modification [41]. Disturbances in mechanical forces including CBF and BM stiffness are often concomitant and recognized as early signs of numerous cerebral disorders [5, 39]. Below, the force ranges and impact on the cerebral vasculature are discussed.

**Table 1:** Mechanical stressors and forces acting at the vasculature. Summary of non-cellular and cellular mechanical forces at the endothelium measured in N/m^2^ (equals 10 dyne/cm^2^ or 1 Pa)

Stressor	Area/cell type	Force range	References
External non-cellular forces			
Wall shear stress	Arteries	0.4–3 N/m^2^	[39, 42–45]
	Arterioles & capillaries	Avg. 1–2 N/m^2^	[46–50]
	Post-capillary venules & veins	< 0.1–4 N/m^2^	[39, 48, 49, 51]
BM stiffness	Arteries & microvessels	~ 50 K to 150 K N/m^2^ or higher	[39, 52–55]
	Venules/venous tissue (outside brain)	~ 3 K-50 K N/m^2^	[54, 56]
Cell–cell interaction forces			
Selectin catch bonds	Leukocytes & ECs	10–30 pN/bond, maybe up to 60 pN	[57–60]
Integrin catch bonds	Leukocytes & ECs	10–30 pN/bond	[61, 62]
Crawling	T cells	up to 0.6 nN/cell	[63]
Diapedesis	Leukocytes	up to 60 nN/cell	[58]
Cell–cell junctions e.g. VE-cadherin and PECAM-1 binding	ECs	10–100 pN/bond (depending on number and type of interacting proteins)	[64, 65]

### External, non-cellular forces

#### Wall shear stress

Wall shear stress (WSS) or endothelial WSS is calculated from the blood viscosity (highly dependent on concentration and mechanical properties of red blood cells) and axial flow velocity gradient at the vessel wall [66]. WSS, arising from laminar or disturbed CBF at the luminal side of BMECs, plays a critical role in maintaining the structure and function of the BBB [38]. Under healthy conditions, the average capillary WSS in the brain ranges from 1 to 2 N/m^2^ [46, 47] (Table 1, Fig. 1A). ECs experience the highest WSS within capillaries and declining hyperbolically to 0.28 N/m^2^ at larger diameter post-capillary venules [48, 67]. This WSS was shown to be required for the differentiation of vascular ECs into a BMEC phenotype [68] using a dynamic in vitro BBB model. In vitro application of capillary-like WSS also inhibited cell proliferation, probably due to induction of the cyclin-dependent kinase inhibitor p21, causing cell cycle arrest [5, 69]. Recent advances in BBB models, which incorporate external mechanical forces such as shear stress and ECM stiffness, have been reviewed elsewhere [70–74].

CBF dynamics strongly alter with age, occlusion and inflammation, whereas both high and low WSS are associated with brain pathology [75]. Aberrant high WSS (> 4 N/m^2^) occurs in hypertension and causes vascular dysfunction through loss of TJ proteins and activation of inflammatory signalling at the BBB [38, 76–78]. Hypertension and cerebrovascular injury can manifest in small vessel disease, which often presents an intermediate state leading to ~ 30% of ischemic stroke and ~ 80% of haemorrhagic intracerebral bleedings [79–82]. In small vessel disease, the stiffened/damaged arteries are unfit to absorb the heightened pulsatility, causing damage to the proximate microvasculature of the brain [83, 84]. In vitro studies confirm that pulsatile WSS on BMECs causes the loss of TJ proteins, reduced P-glycoprotein expression and delocalization of zona occludens-1 (ZO-1) from the cell borders to the cytoplasm and nucleus [77]. Disease severity of small vessel disease is also associated with global and local CBF alterations and recent work has pinpointed distinct region-specific CBF patterns in patients, which were linked to the occurrence of periventricular white matter hyperintensities and total disease burden [85].

During ischemic stroke, blood flow is blocked to a part of the cerebral vascular network, instantly severely lowering CBF in this region, which can be restored upon timely treatment (reperfusion). As a result, BMECs first experience hypoxia and oxidative stress which partially increases their permeability for blood components, fluids, and peripheral cells, causing post-ischemic inflammation [86]. Edema formation follows ischemia-induced sodium and water uptake through shear stress-regulated ion transporter [87]. A number of signalling pathways (including Wnt and Notch pathways) underlying ischemic/reperfusion injury across organs have been recently reviewed [88]. Very low levels of CBF and WSS also prompt increased BMEC proliferation and enhanced BBB permeability through cell layer remodelling [38, 69, 89]. Following reperfusion, BMECs secondly experience enhanced WSS which promotes endothelial-to mesenchymal transition [90]. This (partial) shift to mesenchymal cell properties further enhances BBB permeability [91], which may however reverse at a later timepoint of reperfusion [91]. A recent study, using two-photon imaging to follow vascular leakage post ischemic stroke in mice, showed BBB leakage as early as 30 min after the occlusion, steadily increasing in the hours after (120 min) [92]. Disruption of BBB function from ischemia/reperfusion has been reported as a predictor of poor disease outcome and haemorrhagic transformation in ischemic stroke patients [93].

Hypertension-induced vascular injury correlates with age and increases the risk for AD, yet the CBF is frequently reduced globally and locally in AD patients, which has been suggested as an early marker for AD [20, 94–96]. Similarly, MS patients show reduced CBF in white matter regions, specifically periventricular areas, concomitant with increased BBB permeability [97, 98]. Reduced CBF in MS may be the result of impaired flow-mediated endothelial dilation, both of which are correlated with disease severity and progression [99]. Whereas numerous studies hint towards changes in CBF in age-related and neuroinflammatory diseases [100, 101], the underlying causes and consequences for BMECs and BBB function remain elusive. Especially data on human brain vasculature is missing to fully comprehend different forms of WSS and its effects on the BBB during aging and neuroinflammatory disorders [51].

#### Basement membrane changes

Vascular stiffness is a complex property governed chiefly by vascular reactivity, BM properties, and blood pressure [102]. Here we focus on the BM and its changes in mechanical properties during pathology. The brain endothelial BM separates BMECs from neighbouring pericytes and supports BBB maintenance [103]. The BM is composed of extracellular matrix (ECM) proteins including laminins, nidogens, heparan sulphate proteoglycans, and collagen IV (Fig. 1B). They form a 50–100 nm thick three-dimensional network that interacts with BMECs to hold them in place [104, 105]. BM stiffness is influenced by the composition of ECM proteins, their concentration and elasticity, crosslinking between the proteins and the created intrinsic tension [106]. Degradation through proteases (e.g. matrix metalloproteases), remodelling and (limited) synthesis of ECM proteins can change the degree of membrane stiffness [106].

In vitro studies show that ECs respond to the rigidity of the neighbouring BM by enhanced expression of ECM receptors, including integrins. These cell–matrix contacts are crucial for BMEC identity and BBB function [107, 108]. Consequently, global knockout models for components of the ECM or pharmacological blockage of integrins often demonstrate cerebral bleeds and pre- or postnatal lethality [109, 110]. Growing cells on polyacrylamide hydrogels of varying stiffness showed that cells on soft hydrogels developed a lower density of actin fibres and more rounded nuclei than those grown on stiffer substrates [39, 111]. BM stiffness further cues endothelial progenitor cell differentiation along the arterial-venous axis, as higher stiffness induces arterial EC marker expression via the Ras-Mek pathway [112]. Interestingly, when exposed to physiological WSS, ECs on soft hydrogels displayed tighter cell junctions, more profound elongation, and fewer (RhoA-mediated) cytoskeletal changes, revealing a direct relation between ECM stiffness and WSS [111]. These results show that ECs can directly modulate internal tension in response to external forces by altering the assembly and dynamics of actin fibres and junctional proteins [40, 113].

BM thickening has been linked to a narrowing of the vessel lumen during inflammation, aging and AD [114–118]. Over time, the BM composition shifts towards thicker collagen fibres with enhanced crosslinking, while hyaluronan levels decrease by degradation and elastin elasticity function is reduced [119, 120]. Consequent narrowing of the vessel lumen diameter restricts local CBF, causing lack of sufficient oxygen and nutrient supply for the brain [121, 122]. Prolonged reduced CBF can be further amplified by decreased endothelial-dependent dilation and overactive contraction, e.g. by pericytes evoked through reactive oxygen species or Aβ signalling in ischemic stroke and AD [123, 124]. Moreover, the increase in BM rigidity affects mechanosensitive processes such as angiogenesis [125, 126]. Functional angiogenesis requires a delicate balance between matrix remodelling/degradation and mechanical support of the BM for BMEC adhesion and migration. Age-related changes in BM composition and elasticity result in a lack of vessel support and might be linked to microvessel rarefaction and regression following impaired angiogenesis [127]. Lastly, the endothelial BM is a reservoir for growth factors and signalling crucial for vessel formation. Changing ECM protein composition thus also leads to a dysregulation in growth factor and other ligand availability [127].

In AD pathogenesis, changes in levels of collagen IV and the proteoglycan perlecan occur early on (Braak stage > 2 & ≤ 4) and correlate with parenchymal Aβ plaque deposition in the frontal and temporal cortex, suggesting BM changes in parallel with disease progression [128]. In AD patients with CAA, CAA severity correlates with Aβ_1–42_ and collagen IV abundance in vessels of the frontal cortex [129]. It remains to be investigated if changes in the endothelial BM facilitate vascular Aβ deposition or merely occur concomitant with disease development. In MS, non-invasive pulse wave velocity measurements, a readout for arterial stiffness, are associated with disease duration and severity. Another study showed that increased arterial stiffness correlated with a reduced cognitive processing speed, as assessed via the Symbol Digit Modalities Test [130, 131]. Similar measurements in stroke patients connected arterial stiffness with enlarged perivascular spaces and cerebral microbleeds and revealed vessel stiffness as a potentially more important risk factor than blood pressure for cerebral small vessel disease [132, 133]. Lastly, a meta-analysis demonstrated a negative association between arterial stiffness and cognitive function, together highlighting the importance of vascular fitness in disease pathogenesis and global memory function [134, 135].

Due to their dense network, BMs also create a rate-limiting step for immune cells to enter the perivascular space and the parenchyma, prolonging the migration duration by 3–fourfold [136–138]. Pathological changes in BM composition can facilitate the infiltration of immune cells and cue cell differentiation as previously reviewed elsewhere [139]. Matrikines, peptides released during ECM remodelling, might also affect BMEC phenotype and inflammatory state as suggested for the peripheral endothelium [140, 141]. Ex vivo measurements of BMs have shown a large range of stiffness (Young’s elastic module) from 500 to 4,000,000 N/m^2^ depending on the tissue, but similar to WSS, measuring the stiffness of BMs in vivo remains an ongoing challenge (Table 1) [142].

### Cell–cell interaction forces during transendothelial migration

Immune cell migration across the BBB under homeostasis is limited to a small subset of activated immune cells [143]. Dysfunction of the BBB due to inflammatory and age-related pathology greatly enhances TEM. Immune cell entry further potentiates inflammatory processes within the CNS, fuelling disease progression and cognitive decline [144–146]. In addition to chemical cues, leukocytes prepare for migration by following a stiffness gradient of the underlying substrate, a process termed durotaxis [147, 148]. In the following sections, we highlight the forces acting between BMECs and immune cells during the multi-step process of TEM. Studies on the mechanical forces between BMECs and immune cells have predominantly focused on T cells, which are key players in neuropathological events. However, many of these mechanisms are likely universal to immune cell populations entering the CNS.

#### First steps of engagement of leukocytes with BMECs

**Fig. 2:**
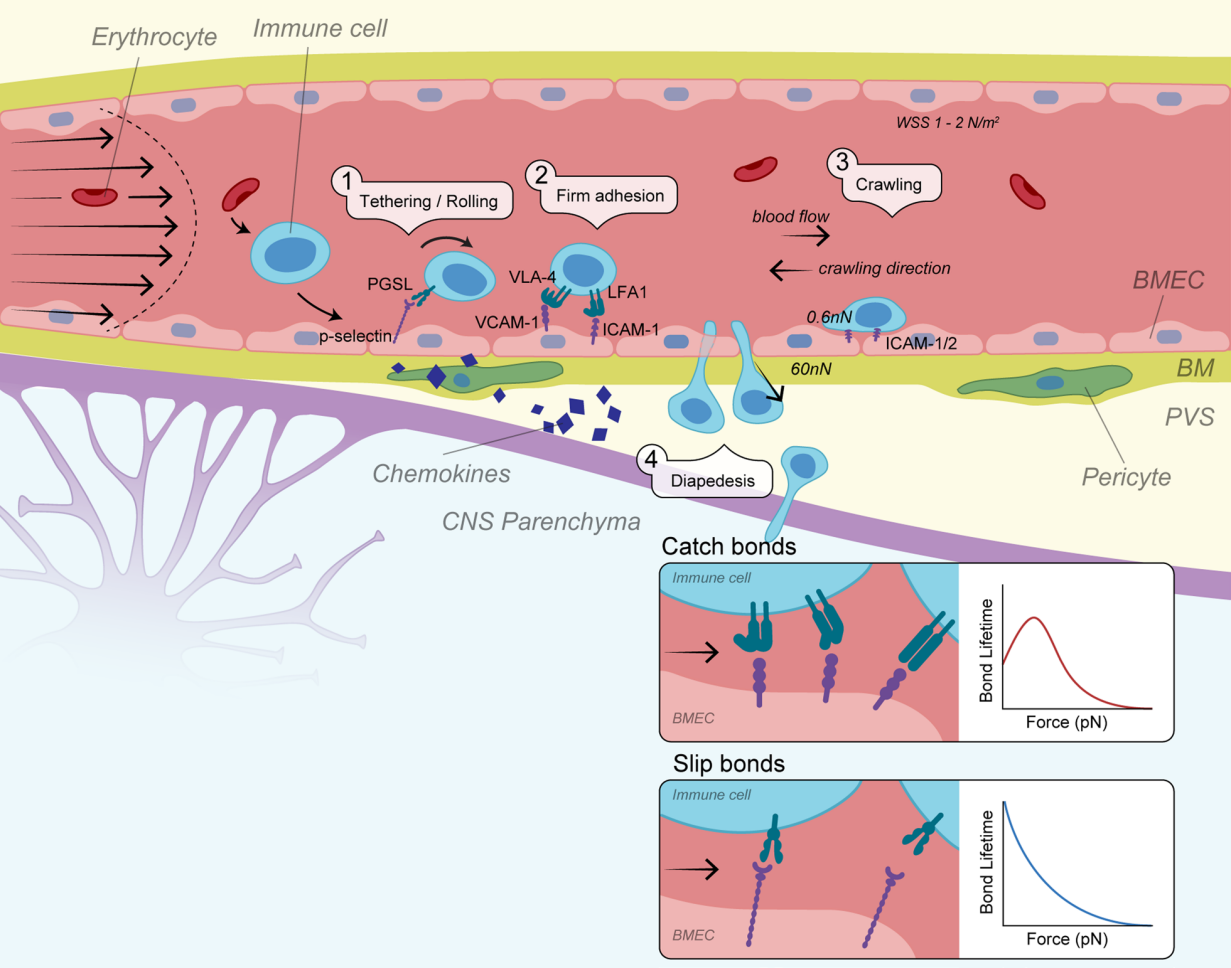
TEM cascade of migratory T cells across the brain endothelium. Upon engagement of catch bonds between selectins/α_4_-integrins and their ligands, T cells start rolling and eventually firmly adhere to the ECs. Cells then crawl against the direction of CBF and probe BMECs for sites permissive for diapedesis. Diapedesis eventually takes place either paracellularly through bi-/tricellular endothelial junctions or transcellularly by inducing the formation of pores within ECs. *WSS* Wall shear stress, *PGSL-1* P-selectin glycoprotein ligand-1, *VLA-4* very-late antigen 4, *VCAM-1* vascular cell adhesion molecule-1, *LFA1* lymphocyte function-associated antigen 1, *ICAM-1* Intercellular adhesion molecule 1, *PVS* perivascular space, *WSS* Wall shear stress, *BM* basement membrane

In order to exit the bloodstream and enter the brain parenchyma, immune cells must come into contact with the vessel wall [149]. Thus, migration primarily takes place at inflamed postcapillary venules where WSS is greatly reduced, increasing the chance of immune cells interacting with the brain endothelium [150]. This is further facilitated by erythrocytes, which are thought to push immune cells to the edges of the blood vessel [149] (Fig. 2).

The first contacts of the leukocyte with the endothelial cells (ECs) are generally made between the family of molecules known as selectins (L-selectin on leukocytes, P- and E-selectin on ECs) and/or α_4_-integrins (such as very-late antigen 4 (VLA-4) and lymphocyte function-associated antigen 1 (LFA-1) on leukocytes), and their respective ligands [151–153]. During inflammatory insults such as in MS, selectin and integrin expression increases in both BMECs and leukocytes [154]. In fact, inhibiting T cell adhesion by blocking the interaction of α_4_-integrins with brain endothelial vascular cell adhesion molecule-1 (VCAM-1) through natalizumab is currently one of the most effective treatments for relapsing–remitting MS [8, 155, 156]. The initial tethering is followed by rolling of the immune cell along the vascular wall with reduced speed. Interestingly, rolling does not seem to be required for successful T cell migration [58, 152, 157]. A direct capture mechanism of T cells, mediated by α_4_-integrins/VCAM-1, has been shown predominantly under non-inflammatory conditions and might be specific for migration into the CNS [158–161].

During rolling, immune cells experience a tangential force caused by WSS, as well as a rotational torque due to their rolling motion, resulting in shear forces dragging on newly formed adhesion complexes [162]. Surprisingly, these forces were found to stabilize adhesive interactions rather than opposing them. This discovery led to the definition of ‘catch bonds’—a bond whose lifetime increases with rising tension, as opposed to ‘slip bonds’ whose interaction is destabilized by force [58, 163–165] (Fig. 2, insert). Catch bond formation is essential for the interaction of selectins and integrins whose lifetime is maximal in a force range of 10–30 pN [57, 61, 62]. Application of lateral force in form of WSS is thought to accelerate the conformational transition of integrins into an extended high-affinity state and thus facilitate immune cell arrest in an outside-in manner [166–168].

Rolling at reduced velocity enables immune cells to probe the endothelial surface for chemokines [58]. These chemokines bind to chemokine receptors on the immune cell surface, delivering a G-protein coupled receptor (GPCR)-mediated inside-out signal to α- and β-integrins. GPCR signalling increases the affinity of integrins for their endothelial ligands via activation of the cytoskeletal adaptors talin and kindlin in the immune cell [169]. Interaction pairs include LFA-1 (T cell) and intercellular adhesion molecule-1/2 (ICAM-1/2) (ECs), as well as VLA-4 (T cell) and VCAM-1 (EC) [152, 153, 167, 170]. This is further facilitated by integrin clustering into focal adhesions and ultimately results in integrin-mediated immune cell arrest, polarization, and firm adhesion on the endothelium [58]. Catch bonds again play a crucial role in this process as integrin activation by chemokines alone is insufficient to stimulate adhesiveness [62, 171]. In summary, the adhesion of immune cells to the post-capillary bed is facilitated by both WSS and intercellular forces, together stabilizing the interaction and allowing for cell migration to occur.

#### T cell crawling on BMECs is directed against the flow

While adhering to the vascular wall, lymphocytes polarize with respect to the blood flow by adopting an elongated morphology with a wide and flat F-actin-rich lamellipodium at the leading edge and a tail-like projected uropod at the trailing edge [160, 172]. Crawling is an active movement facilitated by Rho GTPases [8, 58], involves the formation of additional adhesive contacts, and occurs at a highly reduced velocity of a few µm per minute [173, 174]. Forward propulsion along the endothelium is driven by actin polymerization and myosin II contractility (retrograde flow), which together push the lamellipodium forward, parallel to the surface [58, 63]. Thereby, the actin cytoskeleton generates internal forces of up to 0.6 nN [63], which are transferred to integrins via cytoskeletal adaptors such as talin, increasing the ability to form catch bonds [172]. This actin-talin-integrin linkage, however, is not absolute, leading to varying degrees of slippage [175]. This has been termed a ‘molecular clutch’ and is exploited by the cell to dynamically adjust migration speed and direction in response to changes in substrate adhesiveness and matrix stiffness [175, 176]. Enhanced stiffness, as found in inflammation and aging [148], has been linked to more persistent directional crawling [177] and increased efficiency of transmigration [178].

T cell crawling along the cerebral post-capillary venules is unique in two aspects. Firstly, crawling was observed in vivo to be directed preferentially against the direction of blood flow [8, 138, 152, 179]. This has been linked to the presence of shear forces and the adhesion molecules ICAM-1 and ICAM-2 [161, 172]. T cells can also crawl downstream by engagement of VLA-4 and VCAM-1, but prefer upstream migration under shear rates above 400 s^−1^ [172]. Interestingly, in the absence of VLA-4 or VCAM-1, T cells still adhere and crawl against the flow, but do not maintain directionality after the flow is terminated [161]. VLA-4 thus appears to be required for migratory memory, the capacity to remember directionality, under WSS [180].

Secondly, T cell crawling at the brain post-capillary venules takes place over significantly longer distances, frequently exceeding 150 µm, which is likely due to the unique high barrier integrity rarely allowing for diapedesis even under inflammatory conditions [160]. In contrast to rapid rolling and tethering, crawling takes on average several minutes and rarely up to hours [138, 160, 161]. On primary mouse BMECs, 95% of T cells crawled or transmigrated within half an hour, whereas 5% arrested and remained immobile [160]. Similarly, intravital imaging of autoreactive T cells showed 80–85% of cells crawled and extravasated from the leptomeningeal vessels within 30 min [138]. The purpose and mechanism of preferential crawling against the flow at the BBB still remain elusive, and it has yet to be determined whether this phenomenon is immune cells specific and how frequently this event occurs in other tissues [179].

#### Diapedesis by the path of least resistance

Immune cell migration across BMECs occurs either paracellularly through endothelial junctions or transcellularly by inducing the formation of pore-like structures in the ECs themselves [152]. In search for permissive sites, leukocytes sense endothelial substrate stiffness, mainly defined by their F-actin density [148, 181]. Several factors determine which route will be preferred. High ICAM-1 levels, increased caveolin-1 expression, and chemokine presentation have all been linked to transcellular migration [182–186]. On the other hand, with high stress fibre density and lower levels of inflammation with intermediate ICAM-1 expression might favour paracellular diapedesis [182, 187]. Although increased stiffness appears to facilitate crawling, diapedesis of T cells locally takes place at the path of least mechanical resistance [182, 188]. Transcellular migration thus primarily occurs at the cell periphery, where F-actin density is highly heterogeneous [58, 182, 189]. As multiple forces and chemical signals act in concert, plus in vivo studies are sparse, it is challenging to predict which migration route is preferred in neuroinflammatory conditions like MS, AD, or during aging.

Finally, diapedesis takes place within seconds to minutes [190], during which both leukocytes and ECs undergo drastic cytoskeletal remodelling. Leukocytes adopt an elongated shape and drastically reduce stiffness by inducing actin disassembly and breaking down vimentin intermediate filaments and microtubules [189]. Once the leading edge of the leukocyte has breached the endothelial monolayer, the elongated leukocyte nucleus squeezes through the endothelium with lateral forces in the order of 60 nN to extrude through the narrow space [58]. This is sensed by the endothelial cytoskeleton, which responds with the disassembly of filaments to withstand these forces and allows for transmigration to succeed [189]. Leukocyte squeezing also triggers the formation of an F-actin ring surrounding the migrating immune cell. This ring limits pore size and prevents plasma leakage, allowing the endothelium to preserve its low permeability to macromolecules [189, 191]. Upon successful diapedesis, BMECs respond to the sudden loss of tension and utilize ventral lamellipodia to close the gap [189]. During transmigration, ECs and leukocytes exchange surface proteins [192, 193], which might draw additional immune cells to these sites, creating a hotspot for transmigration [194]. However, it is unclear whether the endothelium returns to its previous state eventually or retains long-lasting imprints at these sites.

The unique structural properties of the BBB would suggest that mechanical force transmission and response are distinct from those observed in other tissues, and might help explain molecular mechanisms directing transcellular or paracellular diapedesis. However, no studies have yet reported mechanical properties of diapedesis across BMECs.

### Mechanosensory elements of endothelial cells

**Fig. 3:**
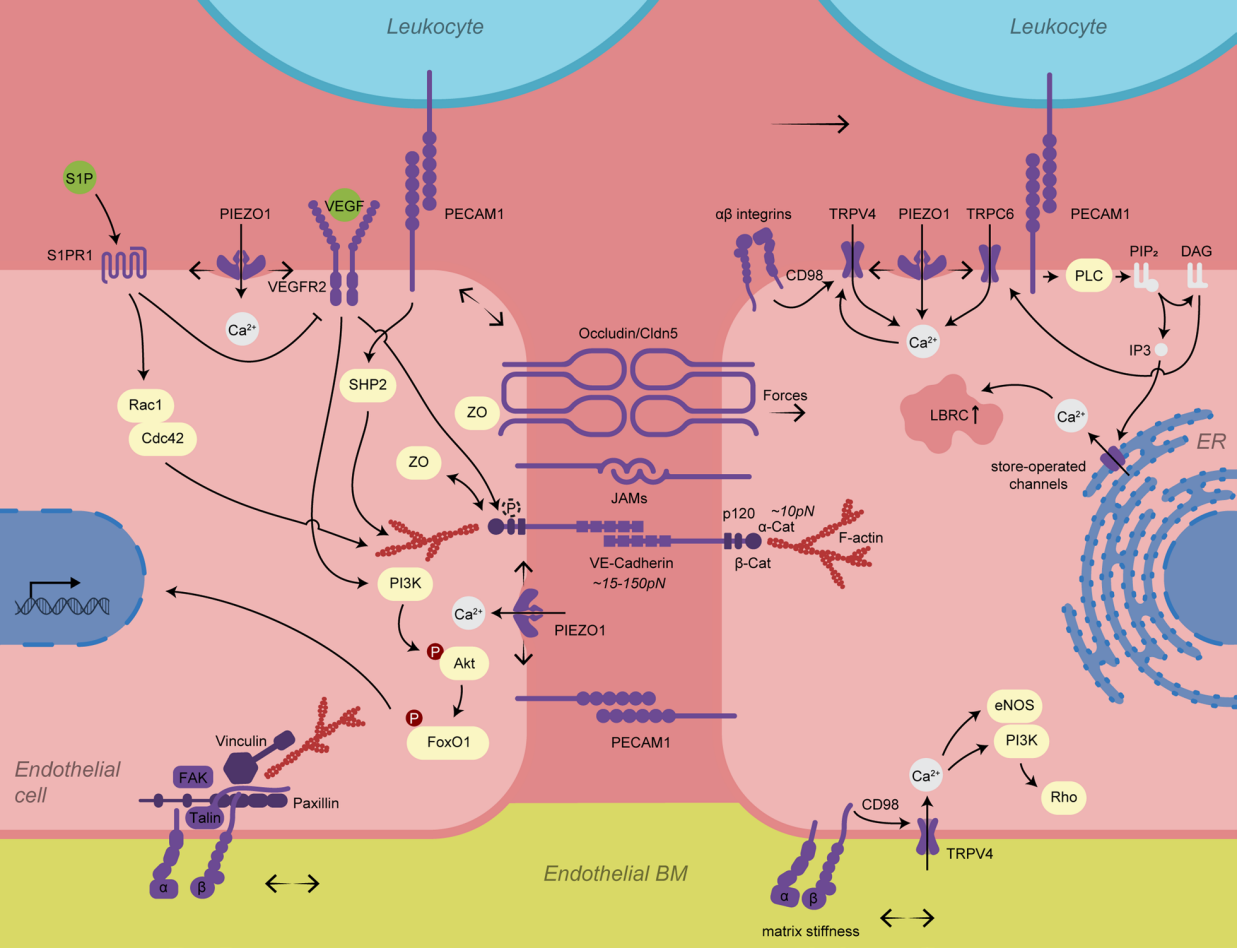
Biomechanical signalling via mechanosensing/sensitive elements at the brain endothelium. Intercellular tension is sensed by junctional molecules such as PECAM-1, VE-cadherin, and catenins. Interactions between PECAM-1, VE-cadherin, Piezo1, S1PR1, and VEGFR2 regulate barrier stability. Integrin signalling transmits ECM stiffness and stretch to the actin cytoskeleton and ion channels. Transient receptor potential (TRP) ion channels may be activated by direct mechanical force transfer from primary mechanosensing components (integrins) or via intermediate proteins (CD98) or cytoskeletal adaptors. Alternatively, they may be activated via biochemical signalling downstream of primary mechanosensing elements, involving synthesis and modification of intracellular messengers (DAG and Ca^2+^). *WSS* wall shear stress, *S1P/S1PR1* sphingosine-1-phosphate receptor 1, *VEGFR2* vascular endothelial growth factor receptor 2, *Rac1* Rac family small GTPase 1, Cdc42 cell division cycle 42, *JAMs* junctional adhesion molecules, *PECAM-1* platelet and endothelial cell adhesion molecule 1, *α/β* cat catenins, *ZO* zonula occludens, *PLC* phospholipase C, *DAG* diacylglycerol, *PIP2* Phosphatidylinositol 4,5-bisphosphate, *Ca*^*2*+^ calcium, *ER* endoplasmic reticulum

Considering the wide variety of mechanical stresses at the BBB, BMECs express a variety of mechanosensory complexes, which convert physical stress into biochemical signals (Fig. 3). Here we focus on cell–cell junction complexes and mechanosignalling through receptors and ion channels of BMECs, highlight their relevance in immune cell migration across the BBB, and changes during inflammation and aging. Importantly, we try to distinguish mechanosensing elements, which were shown to respond to mechanical forces directly, from mechanosensitive players, which may be activated indirectly by force transmission and biochemical signals downstream of primary mechanosensing components or actin fibres.

### Cell–cell junctions

Cell–cell junctions play a crucial role in endothelial barrier function and have thus adapted multiple ways to respond to mechanical forces (Fig. 3) [4, 195]. Formation of stable AJs requires intracellular coupling of VE-cadherin to actomyosin through α-catenin [196, 197]. Both external forces on VE-cadherin and internal forces generated by actomyosin pull α-catenin into an extended conformation, leading to the recruitment of vinculin and other binding partners [4, 196, 197]. Vinculin further stabilizes the unfolded conformation of α-catenin, leading to enhanced cell–cell adhesion and F-actin polymerization [40, 195, 196]. Increasing force strengthens the binding (catch-bond) between the cytoskeleton and junctional partners vinculin, talin, and α-catenin upon formation of dimers/multimers (order of 10 pN), while e.g. a bond between monomeric α-catenin and F-actin disrupts under increasing tension (slip-bond) [198–201]. The actin cytoskeleton of ECs also directly responds to tension via polymerization and interaction with actin filament-associated proteins, leading to stress fibre formation, increased stiffness, junctional changes and activation of focal adhesions [40, 202].

Extracellular detachment forces between VE-cadherin proteins (Ca^2+^-dependent) range from 15 to 150 pN depending on their cumulative interactions and on the applied stretch, which extends their lifetime (catch-bonds) [65, 203]. Cadherins are also thought to be mechanosensitive themselves, acting in concert with PECAM-1 and VEGF receptors to initiate further downstream signalling including PI3K/Akt and integrin/RhoA [6, 40, 64, 76, 204, 205]. Mechanosensing by PECAM-1 regulates force-dependent cellular stiffening [204] and PECAM-1 is critically involved in the regulation of BBB junctional integrity [206], WSS sensation [207], integrin activation, cytoskeletal rearrangement [195, 208] and TEM [209]. Loss of PECAM-1 increases vascular permeability of the BBB, but prevents paracellular transmigration, suggesting that intact (to a certain degree) cell junctions and PECAM-1 mechanosignalling are required for paracellular diapedesis [8, 185, 206]. On the other hand, in pathologies with disturbed WSS, such as atherosclerosis and ischemic stroke, PECAM-1 is increased and defined as a main contributor to atherosclerotic lesions, neutrophil invasion, and post-ischemic neuroinflammation [210, 211].

BM stiffness and WSS regulate the force on focal adhesions and TJs by traction force, hence mechanosensory properties have also been shown for TJ proteins. [6, 212]. Under physiological laminal WSS, mechanosensing via occludin promotes vascular integrity by recruiting further occludin and ZO-1, whereas disturbed flow causes loss of occludin and barrier dysfunction [213]. In addition, the TJ adaptor protein ZO-1 also regulates tension acting on VE-cadherin in response to WSS, similar to the function of α-catenin at AJs [205, 212, 214]. Together these components enable the dynamic response of junctional proteins to mechanical forces, which are required for endothelial barrier formation and maintenance [196, 215], and cell orientation in the direction of flow [195]. In neurological pathologies, both mechanical forces as well as organization and expression of cell–cell junctions change drastically. However, it remains challenging to create a causative relationship between these two components, as other factors, such as cytokine signalling, majorly affect junctional integrity [216].

### Mechanosensitive receptors and ion channels

Both ECs and migrating immune cells display a wide range of receptors and ion channels responding to mechanical stress, although mechanisms of their localization and activation are often not fully understood [217]. Mechanosensitive proteins can be activated by direct stress applied to the lipid bilayer, opening e.g. the ion channel through membrane tension, or via additional linked components outside the membrane [218, 219]. Activated proteins often undergo large conformational changes, such as unfolding of domains in talin, which are reversed upon force dissipation [220, 221]. Of note, translation of mechanical signals into cellular alterations occurs on different time scales e.g. with integrin activation (seconds to minutes) and longer duration for cytoskeletal rearrangements (minutes to hours) [220].

*Tyrosine kinase receptors* on ECs, including vascular endothelial growth factor receptor 2 (VEGFR2) and Tie-2, become phosphorylated under mechanical stretch, which triggers ligand-independent signalling [222, 223]. Importantly, VEGFR2 has multiple phosphorylation sites, which can regulate vascular permeability through VE-cadherin phosphorylation, but also proliferation and migration [224]. As a result, mechanical cues can regulate angiogenesis via VEGFR2-signalling [225]. ECM stiffness regulates the angiogenic potential via yes-associated protein (YAP)-Dll4-Notch1-VEGFR signalling, and promotes pro-angiogenic factors on softer vs. stiffer substrates [226]. YAP/transcriptional coactivator with PDZ-binding motif (TAZ) activation also induces an inflammatory endothelial phenotype under disturbed blood flow, hence inhibition of this pathway has been effective against atherosclerotic lesion development [227, 228]. Exploration of these tightly connected mechanosensitive pathways for therapeutic approaches could reinstate functional angiogenesis in cerebral vessels with stiffer BM and support areas of disturbed or reduced WSS such as in elderly, AD and ischemia [229].

*Mechanosensing GPCRs* change conformation under mechanical stress [230]. For example, endothelial GPR68 is activated by high WSS in small diameter arteries, triggering flow-mediated dilation [231, 232]. Interestingly, GPR68 is also activated by acidosis, a lowered blood pH, which can occur through prolonged lack of oxygen and CO_2_ accumulation [233]. However, CO_2_-induced CBF increase was not changed in mice lacking GPR68, thus the role of GPR68 for H + sensing at cerebral arteries could be less prominent [234, 235]. Sphingosine-1-phosphate receptor 1 (S1PR1) is another mechanosensitive GPCR expressed in BMECs but also widely throughout other tissues [236]. Vascular S1PR1 can be activated by WSS and circulating S1P resulting in vasodilation and maintenance of endothelial barrier stability [237]. Of note, S1PR modulators, such as fingolimod (S1PR1,3,4,5 modulator) and siponimod (S1PR1,5), are approved as disease-modifying treatment in MS, and tested in clinical trials for ischemic and hemorrhagic stroke [238, 239]. Fingolimod also reduces VCAM-1 expression and stabilizes claudin-5 levels on BMECs [240]. In line with SP1R1s mechanosensitive functions in vasodilation and vascular network maintenance, prolonged treatment with fingolimod resulted in hypertension in mice [241] and patients with chronic inflammatory demyelinating polyradiculoneuropathy [242]. In conclusion, mechanosensitive GPCRs present a promising target group for multiple CNS diseases, however, their widespread expression and functional spectrum may limit therapeutic potential.

*Mechanosensitive ion channels* are primarily cation permeable and regulate ion flux to initiate Ca^2+^ signalling, alter the membrane potential, and regulate Mg^2+^ homeostasis [36, 40, 243]. Due to their rapid action and the fast propagation of Ca^2+^ waves, ion channel activity is usually among the first steps in mechanical signalling [40, 244]. This Ca^2+^ signalling is critical for maintaining the low permeability of the BBB [245], whereas dysregulation of intracellular Ca^2+^ due to high mechanical stress or pathological conditions can compromise BBB integrity [245–248]. For example, Ca^2+^ influx through the nonselective cation channel Piezo1 is involved in flow-induced cell alignment, vasodilation of cerebral capillaries and angiogenesis [40, 249–251]. Overstimulation of Piezo1 due to disturbed flow conditions in atherosclerosis and hypertension, however, causes the breakdown of AJs, actin remodelling and inflammation [78, 252–255]. The underlying mechanism is a direct connection between Piezo1 and VE-cadherin as well as PECAM-1 regulating junction integrity [256].

Mechanosensitive ion channels also include the diverse family of transient receptor potential (TRP) ion channels, which mediate large parts of the mechanical stress response experienced by BMECs. They fulfil a multitude of functions in immune cell migration and barrier function and are involved in both progression and recovery of several neurological and vascular-related pathologies [35, 36, 257]. The following section will highlight the functional diversity of this family of ion channels.

### Mechanosensitive TRP channels

TRP channels are widely expressed throughout the CNS and are involved in cellular response to a variety of external cues, including mechanical signals [258] (Table 2). Based on sequence homology, they are grouped into six subfamilies termed canonical (TRPC1-7), vanilloid (TRPV1-6), ankyrin (TRPA1), polycystic (TRPP1-3), melastatin (TRPM1-8) and mucolipin (TRPML1-3) [36]. TRP channels can directly or indirectly trigger Ca^2+^ signalling [40, 245] and can also be Ca^2+^-regulated themselves via Ca^2+^-calmodulin or direct Ca^2+^ binding [40]. Although TRP channels are commonly involved in mechanosignalling cascades, likely none of them are mechanosensing themselves [259]. Mechanisms for direct mechanical activation, such as deformation due to membrane stretch, have been proposed, but lack sufficient evidence [260]. TRP channels are thus most likely activated by mechanical transduction via interaction with mechanosensing proteins, such as β_1_-integrins and the glycocalyx [40, 261]. Alternatively, force transmission may activate TRP channels via intermediary membrane proteins and the cytoskeleton [40]. Table 2 provides an overview of known mechanosensitive TRP channels and associated upstream components. For several TRP subunits mechanosensitive properties have not been shown specifically for vascular ECs, but are likely based on findings in other cell types (indicated by superscript **a** in Table 2).

**Table 2:** Mechanosensitive TRP channels of vascular ECs and leukocytes

Channel	Cell type	Upstream components	Reference(s)
TRPA1	BMECs, T cells, B cells, macrophages	[Ca^2+^]_i_	[262–264]
TRPC1	cBAECs, mBMECs, T cells, B cells, macrophages, neutrophils	PLC, DAG, [Ca^2+^]_i_	[246, 265–268]
TRPC3^a^	cBAECs, mBMECs, T cells, B cells, macrophages, neutrophils	PLC, IP_3_	[246, 265, 266, 269–272]
TRPC5^a^	cBAECs, mBMECs, macrophages	PLC, PIP_2_	[246, 266, 273, 274]
TRPC6	cBAECs, macrophages, neutrophils	DAG, Gα_q_, PECAM-1	[266, 275–278]
TRPM4^a^	BMECs, T cells, macrophages	IP_3_, Ca^2+^-calmodulin	[279–282]
TRPM7^a^	HUVECs, T cells, macrophages		[265, 283]
TRPP1	HUVECs		[284]
TRPP2	BMECs	glycocalyx, IP_3_	[247, 261]
TRPV1^a^	BMECs, T cells, macrophages, neutrophils		[285–288]
TRPV2	BMECs, T cells, B cells, macrophages, neutrophils	phosphatidylinositol-3 kinase, Akt	[285, 286, 288–290]
TRPV4	BMECs, T cells, macrophages, neutrophils	β_1_-integrins, CD98, Piezo1, PLC, PIP_2_, Ca^2+^-calmodulin, arachidonic acid metabolites	[78, 285, 286, 291–295]
TRPV5^a^	T cells, granulocytes		[296]
TRPV6^a^	Jurkat T cells, granulocytes		[297]

Expression of mechanosensitive TRP channels in human primary vascular ECs (BMECs where data available) and leukocytes, and associated suggested upstream signalling components; BMECs–(human) brain microvascular endothelial cells; [Ca^2+^]_i_–intracellular Ca^2+^; cBAECs–cultured brain artery endothelial cells; DAG–diacylglycerol; HUVECs–human umbilical cord endothelial cells; mBMECs–mouse brain microvessel endothelial cells; PIP_2_–phosphatidylinositol-4,5-biphosphate; PLC- phospholipase C

^a^Mechanosensitive properties derived from cell types other than ECs or leukocytes

#### TRPV4 is a key mediator of mechanical signalling

TRPV4 is a non-selective cation channel triggering downstream signalling via influx of extracellular Ca^2+^ [298] and is involved in mechanosignalling of various cell types, including BMECs [286], T cells [285], neutrophils [294] and macrophages [295]. TRPV4 has been proposed to react to diverse mechanical forces such as WSS [78, 299–302], circumferential stretch [303, 304], stiffness [305, 306], osmotic swelling [298, 307], shrinkage and surface expansion [298].

Endothelial TRPV4 is an important regulator for flow-induced vasodilation [299, 302], cell volume [243, 293], cytoskeletal remodelling and barrier function [248, 308, 309]. TRPV4 activation can occur within milliseconds in focal adhesions of ECs by force transfer from mechanosensing β_1_-integrins via CD98 to TRPV4 [292, 310]. TRPV4 activation by β_1_-integrins might explain why high ECM stiffness, such as during inflammation or aging, causes endothelial dysfunction in a pathway that involves TRPV4-mediated Ca^2+^ signalling [305]. TRPV4 is also involved in flow-induced vasodilation and cytoskeletal remodelling via Piezo1. Upon mechanical stimulation, Piezo1 causes a local increase in intracellular Ca^2+^ and activates TRPV4 via Ca^2+^-calmodulin binding to its C-terminal calmodulin binding site [78, 298, 311]. TRPV4 then triggers a more prolonged Ca^2+^ signal, which leads to Piezo1-mediated disassembly of AJs under disturbed blood flow [78]. TRPV4 inhibition in a mouse model of haemorrhagic stroke reduced BBB dysfunction, likely due to reduced Piezo1 signalling [309, 312]. These findings suggest a regulatory role of TRPV4 in vascular permeability, which contributes to BBB disruption under altered mechanical conditions [308, 309]. This role in BBB function and fast activation via β_1_-integrins render TRPV4 a potential contributor to immune cell crawling and diapedesis [248]. It remains to be determined at what stage of the migration cascade TRPV4-mediated signalling occurs.

#### TRPC channels form mechanosensitive homomeric and heteromeric channels

Channels of the TRPC subfamily are involved in stretch and shear stress sensing of ECs and migrating immune cells [313, 314]. Although the expression of TRPCs in primary human BMECs has not explicitly been shown, their frequent expression in other human endothelial tissue [315] would suggest that they are also common at the BBB. This is further supported by findings of TRPC expression in cultured brain artery ECs (cBAECs) [266] and mouse brain microvessel ECs (mBMECs) [246, 316]. All TRPC channel complexes in humans can be activated by PLC-mediated hydrolysis of PIP_2_ [278, 317]. This can directly lead to TRPC channel opening by binding of diacylglycerol (DAG) or removal of PIP_2_-dependent inhibition [317–319]. Alternatively, IP_3_ may travel through the cytoplasm, bind to its receptor and trigger the release of Ca^2+^ from intracellular stores, which in turn activates TRPC channels by Ca^2+^ or Ca^2+^-calmodulin binding [35, 260, 317, 320]. Activation by mechanical stress might thus involve mechanosensing GPCRs activating G_q/11_ proteins, which in turn stimulate PLC and lead to TRPC channel opening further downstream [313].

Whereas TRPC1 is essential for cell migration and polarization [203, 243, 313], TRPC6 plays an important regulatory role in barrier-forming tissue [40, 209]. Endothelial TRPC6 channels mediate the intracellular Ca^2+^ signalling to allow leukocyte diapedesis [189, 209]. Weber et al. [209] showed that TRPC6-deficient ECs prevent paracellular diapedesis, whereas selective activation of TRPC6 by a DAG analogue allows for transmigration even when PECAM-1 is blocked. These results suggest that TRPC6 is activated downstream of PECAM-1-mediated mechanosensing and is required for the cytoskeletal adaptations in ECs enabling leukocytes passing through. The same study also showed that TRPC6 channels accumulate around PECAM-1 and promote translocation of intracellular stores of PECAM-1 and other adhesion molecules to the site of diapedesis [209]. TRPC6-induced Ca^2+^ signalling is likely also responsible for the formation of the F-actin ring surrounding migrating immune cells [189]. Under pro-inflammatory and disturbed flow conditions, TRPC6 expression and activation are elevated independently of diapedesis, leading to EC contraction and increased vascular permeability [321, 322].

TRPC channels function both as homomeric complexes and in heteromeric combinations, which differ in terms of activation characteristics, sensitivity and cation selectivity [40, 314, 323]. For example, vasorelaxation by ECs in response to high WSS is in part realized by heteromeric complexes of TRPC1 and TRPV4 [40, 314, 324]. The formation of heteromers might explain the multiple and often overlapping functionalities of TRP subunits [40, 314] and may be a major reason why most TRP knockouts in animal models do not show a detrimental phenotype [309]. How the formation of these complexes is regulated and how their functionalities differ from their homomeric counterparts requires further investigation.

#### TRPP, TRPM and TRPA1 channels regulate vascular tone, permeability and function

TRPP1 and TRPP2, also known as Polycytin-1 and −2, are nonselective cation channels involved in flow and pressure sensing of vascular ECs [323, 325]. Both subunits accumulate at primary cilia where they regulate vascular tone in response to WSS by inducing nitric oxide production [284, 325, 326]. Upon mechanical injury of BMECs, TRPP2 is involved in stress fibre formation and cytoskeletal remodelling leading to BBB dysfunction [245, 247]. Interestingly, TRPP2 may only be mechanosensitive in combination with other TRP subunits, including TRPP1, TRPC1 and TRPV4, once again highlighting the relevance of heteromeric TRP complexes [323, 325]. Accordingly, pressure sensing by ECs was shown to be dependent on relative expression of TRPP1 and TRPP2 [325], and heteromeric complexes of TRPV4 and TRPP2 are much more sensitive to laminar WSS than homomeric TRPV4 channels [327]. Changing composition of heteromeric TRP channels might thus present a useful way by which cells regulate their sensitivity to mechanical stress.

TRPM channels are widely expressed in the brain and are involved in many neurological disorders [35]. TRPM4 was shown to be activated by membrane stretch in vascular smooth muscle cells [280], but its role in BMECs still remains elusive. In vascular ECs, TRPM4 associates with Sulfonylurea receptor 1, which is upregulated in BMECs after ischemic stroke [35]. This causes continuous Na^+^ influx and can lead to cell death, disintegration of capillaries and secondary bleeding [35, 328]. TRPM7 is another potentially mechanosensitive channel, which is activated by shear stress in fibroblasts [329], vascular smooth muscle cells [330] and mesenchymal stem cells [331]. Knockdown of TRPM7 in human umbilical cord ECs (HUVECs) prevents adhesion of ECs and promotes their growth and proliferation, suggesting a regulatory role in cell differentiation [283].

TRPA1 is involved in vasodilation of BMECs [262, 332] and is also expressed in T cells, where it promotes immune functions in response to mechanical stress and is implicated with several inflammatory conditions [263, 264]. TRPA1 can be activated by Ca^2+^ influx [35] and may even be mechanosensing itself. Although a recent study showed that TRPA1 in reconstituted proteoliposomes is insensitive to membrane stretch [259], another study revealed that single-channel currents of TRPA1 in artificial lipid bilayers respond to mechanical stretch in a pressure-dependent way [333]. This discrepancy might be explained by its redox state, as mechanosensing of TRPA1 was abolished under reducing conditions [333]. Clearly, TRP channel activation and function are not yet fully explained. Further studies should focus on the effects of redox and other environmental conditions on TRP subunits, as these might help to better explain their activation, function and changes under pathological conditions.

## Discussion and future perspectives

Mechanical stressors are crucial regulators of BBB function. Physiological WSS and BM stiffness support the formation of AJs and TJs and suppress inflammatory signalling, whereas alterations under pathological conditions lead to enhanced vascular permeability, endothelial dysfunction, reduced vascular reactivity and facilitate leukocyte infiltration. In this review, we took a holistic view of the various aspects of mechanical forces and mechanosensing at BMECs in different neuropathologies, which open new future intervention avenues.

Increasing evidence suggests that mechanisms of immune cell infiltration are not conserved across the body and depend on the tissue and immune cell type involved. Such cell-type-specific events offer great opportunities for therapeutic interventions. In MS, for example, Th1, Th17 and CD8^+^ T cells are thought to promote inflammation in the CNS, whereas Th2 cells are associated with a protective effect [334, 335]. Th2 cells show lower ability to form tethers and slings and thus egress to a much lesser extent under high WSS [336, 337]. T cell subtype-dependent behaviour has also been observed for crawling [338] and diapedesis [185], but the underlying molecular mechanisms governing these differences remain mostly undescribed [189]. Closer examination of the molecular players driving cell-type-specific events throughout migration could reveal novel therapeutic targets for the treatment of MS and other neuroinflammatory or age-related conditions.

TEM is controlled by a range of mechanosensory elements on both migrating leukocytes and the endothelium. It is not always clear whether certain mechanosensory proteins adapt to pathological conditions to maintain vascular integrity, or whether these conditions throw mechanosignalling events off-balance and thereby contribute to pathological hallmarks. For example, PECAM-1 mediates vasodilation to alleviate high shear stress and might be involved in restoring the BBB after disruption in MS [206, 339], but also contributes to immune cell infiltration and inflammation in atherosclerosis, stroke and MS [211, 339, 340]. Moreover, disturbed flow and inflammation frequently affect expression and localization of endothelial proteins, further altering their response [341]. Decreased surface expression of VE-cadherin during inflammation increases permeability and reduces the ability of ECs to respond to WSS [342]. To arrive at a better understanding of pathological effects on the mechanosensory response, future research will require a more detailed exploration of mechanosensing and -sensitive proteins during neuroinflammation and aging.

Mechanosensitive TRP channels fulfil a wide range of functions and thus offer a plethora of potential drug interventions. Tremendous effort has been put into the development of specific TRP channel agonists and antagonists as drug targets for a range of diseases, including MS, Alzheimer’s disease and stroke [343]. A recent study from our lab explores specifically the potential of TRPV4 inhibition to reduce leukocyte migration across the BBB in MS, however without integrating WSS or BM stiffness [248]. Although numerous potential compounds have entered clinical trials, several of them were eventually withdrawn due to lacking effectiveness and a variety of adverse effects [343, 344]. To effectively use TRP channels for drug discovery, a better understanding of channel expression and activity during pathology is required.

In conclusion, biomechanical signalling is an essential component of BBB function, yet many questions remain open on how these signals change during neuropathology. Continuing research in this field will provide new insights into the cumulative effects of mechanical alterations in WSS, BM stiffness and cell–cell interactions during immune cell migration. Ideally, such understanding would lead to the development of novel drug candidates selectively altering mechanosensory properties of immune cell subsets while leaving other functionalities unaffected, thus counteracting the problem of multifunctionality.

## Data Availability

No datasets were generated or analysed during the current study.

## Abbreviations

Aβ: Amyloid beta
ACA: Anterior cerebral artery
AD: Alzheimer’s disease
AJ: Adherens junction
BAEC: Brain artery endothelial cells
BBB: Blood–brain barrier
BM: Basement membrane
BMEC: Brain microvascular endothelial cell
CAA: Cerebral amyloid angiopathy
CBF: Cerebral blood flow
CCA: Common carotid artery
Cdc42: Cell division cycle 42
CNS: Central nervous system
CSF: Cerebrospinal fluid
DAG: Diacylglycerol
EC: Endothelial cell
ECM: Extracellular matrix
WSS: Wall shear stress
GPCR: G-protein coupled receptor
HSPGs: Heparan sulfate proteoglycans
HUVEC: Human umbilical cord endothelial cells
ICA: Internal carotid artery
ICAM-1: Intercellular adhesion molecule 1
IJV: Internal jugular vein
IP_3_: Inositol 1,4,5-trisphosphate
JAM: Junctional adhesion molecules
LBRC: Lateral border recycling compartment
LFA1: Lymphocyte function-associated antigen 1
mBMEC: Mouse brain microvessel endothelial cells
MRI: Magnetic resonance imaging
MS: Multiple sclerosis
NFkB: Nuclear factor kappa B subunit
PECAM-1: Platelet and endothelial cell adhesion molecule 1
PIP_2_: Phosphatidylinositol-4,5-biphosphate
PLC: Phospholipase C
Rac1: Rac family small GTPase 1
ROS: Reactive oxygen species
S1P/S1PR1: Sphingosine-1-phosphate receptor 1
TAZ: Transcriptional coactivator with PDZ-binding motif
TEM: Transendothelial migration
TJ: Tight junction
TRP: Transient receptor potential
VCAM-1: Vascular cell adhesion molecule-1
VEGFR2: Vascular endothelial growth factor receptor 2
VLA-4: Very-late antigen 4
YAP: Yes-associated protein
ZO: Zonula occludens

## References

[1] Obermeier B, Verma A, Ransohoff RM. The blood–brain barrier. In: Pittock SJ, Vincent A, editors. Handbook of clinical neurology. Amsterdam: Elsevier; 2016. p. 39–59. 10.1016/B978-0-444-63432-0.00003-7 27112670

[2] Varatharaj A, Galea I. The blood-brain barrier in systemic inflammation. Brain Behav Immun. 2017;60:1–12. 26995317 10.1016/j.bbi.2016.03.010

[3] Zlokovic BV. The blood-brain barrier in health and chronic neurodegenerative disorders. Neuron. 2008;57(2):178–201. 18215617 10.1016/j.neuron.2008.01.003

[4] Angulo-Urarte A, Van Der Wal T, Huveneers S. Cell-cell junctions as sensors and transducers of mechanical forces. Biochimica et Biophysica Acta (BBA) - Biomembranes. 2020;1862(9):183316. 32360073 10.1016/j.bbamem.2020.183316

[5] Cucullo L, Hossain M, Puvenna V, Marchi N, Janigro D. The role of shear stress in blood-brain barrier endothelial physiology. BMC Neurosci. 2011;12:40. 21569296 10.1186/1471-2202-12-40 PMC3103473

[6] Conway DE, Breckenridge MT, Hinde E, Gratton E, Chen CS, Schwartz MA. Fluid shear stress on endothelial cells modulates mechanical tension across VE-cadherin and PECAM-1. Curr Biol. 2013;23(11):1024–30. 23684974 10.1016/j.cub.2013.04.049 PMC3676707

[7] Profaci CP, Munji RN, Pulido RS, Daneman R. The blood-brain barrier in health and disease: Important unanswered questions. J Exp Med. 2020. 10.1084/jem.20190062. 32211826 10.1084/jem.20190062 PMC7144528

[8] Marchetti L, Engelhardt B. Immune cell trafficking across the blood-brain barrier in the absence and presence of neuroinflammation. Vasc Biol. 2020;2(1):H1-h18. 32923970 10.1530/VB-19-0033 PMC7439848

[9] Castro Dias M, Coisne C, Lazarevic I, Baden P, Hata M, Iwamoto N, et al. Claudin-3-deficient C57BL/6J mice display intact brain barriers. 2019. Sci Rep. 10.1038/s41598-018-36731-3. 10.1038/s41598-018-36731-3 PMC6338742 30659216

[10] Iwamoto N, Higashi T, Furuse M. Localization of angulin-1/LSR and tricellulin at tricellular contacts of brain and retinal endothelial cells in vivo. Cell Struct Funct. 2014;39(1):1–8. 24212375 10.1247/csf.13015

[11] Arts JJG, Mahlandt EK, Schimmel L, Grönloh MLB, van der Niet S, Klein B, et al. Endothelial focal adhesions are functional obstacles for leukocytes during basolateral crawling. Front Immunol. 2021;12:667213. 34084168 10.3389/fimmu.2021.667213 PMC8167051

[12] Minagar A, Alexander JS. Blood-brain barrier disruption in multiple sclerosis. Mult Scler. 2003;9(6):540–9. 14664465 10.1191/1352458503ms965oa

[13] Alexander JS, Zivadinov R, Maghzi A-H, Ganta VC, Harris MK, Minagar A. Multiple sclerosis and cerebral endothelial dysfunction: mechanisms. Pathophysiology. 2011;18(1):3–12. 20663648 10.1016/j.pathophys.2010.04.002

[14] Pradeepkiran JA, Baig J, Islam MA, Kshirsagar S, Reddy PH. Amyloid-β and phosphorylated tau are the key biomarkers and predictors of Alzheimer’s disease. Aging Dis. 2024. 10.1433/AD.2024.0286. 38739937 10.14336/AD.2024.0286 PMC11964437

[15] Mehta RI, Mehta RI. The vascular-immune hypothesis of Alzheimer’s disease. Biomedicines. 2023. 10.3390/biomedicines11020408. 36830944 10.3390/biomedicines11020408 PMC9953491

[16] Greenberg SM, Bacskai BJ, Hernandez-Guillamon M, Pruzin J, Sperling R, van Veluw SJ. Cerebral amyloid angiopathy and Alzheimer disease—one peptide, two pathways. Nat Rev Neurol. 2020;16(1):30–42. 31827267 10.1038/s41582-019-0281-2 PMC7268202

[17] Ho VS, Cenzer IS, Nguyen BT, Lee SJ. Time to benefit for stroke reduction after blood pressure treatment in older adults: a meta-analysis. J Am Geriatr Soc. 2022;70(5):1558–68. 35137952 10.1111/jgs.17684 PMC9106841

[18] Cheng X, Potenza DM, Brenna A, Ajalbert G, Yang Z, Ming XF. Aging increases hypoxia-induced endothelial permeability and blood-brain barrier dysfunction by upregulating arginase-II. Aging Dis. 2024. 10.1433/AD.2023.1225. 38300641 10.14336/AD.2023.1225 PMC11567255

[19] Binnewijzend MA, Benedictus MR, Kuijer JP, van der Flier WM, Teunissen CE, Prins ND, et al. Cerebral perfusion in the predementia stages of Alzheimer’s disease. Eur Radiol. 2016;26(2):506–14. 26040647 10.1007/s00330-015-3834-9 PMC4712243

[20] Hays CC, Zlatar ZZ, Wierenga CE. The utility of cerebral blood flow as a biomarker of preclinical Alzheimer’s disease. Cell Mol Neurobiol. 2016;36(2):167–79. 26898552 10.1007/s10571-015-0261-z PMC5278904

[21] Shimizu H, Ghazizadeh M, Sato S, Oguro T, Kawanami O. Interaction between beta-amyloid protein and heparan sulfate proteoglycans from the cerebral capillary basement membrane in Alzheimer’s disease. J Clin Neurosci. 2009;16(2):277–82. 19091577 10.1016/j.jocn.2008.04.009

[22] Howe MD, McCullough LD, Urayama A. The role of basement membranes in cerebral amyloid angiopathy. Front Physiol. 2020;11:601320. 33329053 10.3389/fphys.2020.601320 PMC7732667

[23] Chabriat H, Joutel A, Dichgans M, Tournier-Lasserve E, Bousser MG. Cadasil. Lancet Neurol. 2009;8(7):643–53. 19539236 10.1016/S1474-4422(09)70127-9

[24] Joutel A, Monet-Leprêtre M, Gosele C, Baron-Menguy C, Hammes A, Schmidt S, et al. Cerebrovascular dysfunction and microcirculation rarefaction precede white matter lesions in a mouse genetic model of cerebral ischemic small vessel disease. J Clin Invest. 2010;120(2):433–45. 20071773 10.1172/JCI39733 PMC2810078

[25] Joutel A, Haddad I, Ratelade J, Nelson MT. Perturbations of the cerebrovascular matrisome: a convergent mechanism in small vessel disease of the brain? J Cereb Blood Flow Metab. 2016;36(1):143–57. 25853907 10.1038/jcbfm.2015.62 PMC4758555

[26] Saji N, Toba K, Sakurai T. Cerebral small vessel disease and arterial stiffness: tsunami effect in the brain? Pulse. 2016;3(3–4):182–9. 27195239 10.1159/000443614 PMC4865071

[27] Weller RO, Subash M, Preston SD, Mazanti I, Carare RO. Perivascular drainage of amyloid-beta peptides from the brain and its failure in cerebral amyloid angiopathy and Alzheimer’s disease. Brain Pathol. 2008;18(2):253–66. 18363936 10.1111/j.1750-3639.2008.00133.x PMC8095597

[28] Govindpani K, McNamara LG, Smith NR, Vinnakota C, Waldvogel HJ, Faull RL, Kwakowsky A. Vascular dysfunction in Alzheimer’s disease: a prelude to the pathological process or a consequence of it? J Clin Med. 2019. 10.3390/jcm8050651. 31083442 10.3390/jcm8050651 PMC6571853

[29] Katritsis D, Kaiktsis L, Chaniotis A, Pantos J, Efstathopoulos EP, Marmarelis V. Wall shear stress: theoretical considerations and methods of measurement. Prog Cardiovasc Dis. 2007;49(5):307–29. 17329179 10.1016/j.pcad.2006.11.001

[30] Zabrodskaya Y, Paramonova N, Litovchenko A, Bazhanova E, Gerasimov A, Sitovskaya D, et al. Neuroinflammatory dysfunction of the blood-brain barrier and basement membrane dysplasia play a role in the development of drug-resistant epilepsy. Int J Mol Sci. 2023. 10.3390/ijms241612689. 37628870 10.3390/ijms241612689 PMC10454729

[31] Greiner T, Kipp M. What guides peripheral immune cells into the central nervous system? Cells. 2021. 10.3390/cells10082041. 34440810 10.3390/cells10082041 PMC8392645

[32] Hu D, Weiner HL. Unraveling the dual nature of brain CD8+ T cells in Alzheimer’s disease. Mol Neurodegener. 2024;19(1):16. 38355649 10.1186/s13024-024-00706-y PMC10865558

[33] Smolders J, Heutinck KM, Fransen NL, Remmerswaal EBM, Hombrink P, Ten Berge IJM, et al. Tissue-resident memory T cells populate the human brain. Nat Commun. 2018;9(1):4593. 30389931 10.1038/s41467-018-07053-9 PMC6214977

[34] Ortiz GG, Pacheco-Moisés FP, Macías-Islas M, Flores-Alvarado LJ, Mireles-Ramírez MA, González-Renovato ED, et al. Role of the blood-brain barrier in multiple sclerosis. Arch Med Res. 2014;45(8):687–97. 25431839 10.1016/j.arcmed.2014.11.013

[35] Huang Q, Wang X, Lin X, Zhang J, You X, Shao A. The role of transient receptor potential channels in blood-brain barrier dysfunction after ischemic stroke. Biomed Pharmacother. 2020;131:110647. 32858500 10.1016/j.biopha.2020.110647

[36] Earley S, Brayden JE. Transient receptor potential channels in the vasculature. Physiol Rev. 2015;95(2):645–90. 25834234 10.1152/physrev.00026.2014 PMC4551213

[37] Vanlandewijck M, He L, Mäe MA, Andrae J, Ando K, Del Gaudio F, et al. A molecular atlas of cell types and zonation in the brain vasculature. Nature. 2018;554(7693):475–80. 29443965 10.1038/nature25739

[38] Wang X, Xu B, Xiang M, Yang X, Liu Y, Liu X, Shen Y. Advances on fluid shear stress regulating blood-brain barrier. Microvasc Res. 2020;128:103930. 31639383 10.1016/j.mvr.2019.103930

[39] Gray KM, Stroka KM. Vascular endothelial cell mechanosensing: new insights gained from biomimetic microfluidic models. Semin Cell Dev Biol. 2017;71:106–17. 28633977 10.1016/j.semcdb.2017.06.002

[40] Fels B, Kusche-Vihrog K. It takes more than two to tango: mechanosignaling of the endothelial surface. Pflügers Arch Eur J Physiol. 2020;472(4):419–33. 32239285 10.1007/s00424-020-02369-2 PMC7165135

[41] Zhang X, Kim T-H, Thauland TJ, Li H, Majedi FS, Ly C, et al. Unraveling the mechanobiology of immune cells. Curr Opin Biotechnol. 2020;66:236–45. 33007634 10.1016/j.copbio.2020.09.004 PMC7524653

[42] Samady H, Eshtehardi P, McDaniel MC, Suo J, Dhawan SS, Maynard C, et al. Coronary artery wall shear stress is associated with progression and transformation of atherosclerotic plaque and arterial remodeling in patients with coronary artery disease. Circulation. 2011;124(7):779–88. 21788584 10.1161/CIRCULATIONAHA.111.021824

[43] Hademenos GJ, Massoud TF. Biophysical mechanisms of stroke. Stroke. 1997;28(10):2067–77. 9341720 10.1161/01.str.28.10.2067

[44] Kamimura T, Aoki S, Nezu T, Eto F, Shiga Y, Nakamori M, et al. Association between carotid wall shear stress-based vascular vector flow mapping and cerebral small vessel disease. J Atheroscler Thromb. 2023;30(9):1165–75. 36328567 10.5551/jat.63756 PMC10499442

[45] Rastegari K, Mokhtari-Dizaji M, Harirchian MH, Hashemi H, Ayoobi Yazdi N, Saberi H. Biomechanical changes of the common carotid artery and internal jugular vein in patients with multiple sclerosis. Ultrasonography. 2023;42(1):100–10. 36503209 10.14366/usg.22053 PMC9816705

[46] Kamiya A, Bukhari R, Togawa T. Adaptive regulation of wall shear stress optimizing vascular tree function. Bull Math Biol. 1984;46(1):127–37. 6713148 10.1007/BF02463726

[47] Wong AD, Ye M, Levy AF, Rothstein JD, Bergles DE, Searson PC. The blood-brain barrier: an engineering perspective. Front Neuroeng. 2013;6:7. 24009582 10.3389/fneng.2013.00007 PMC3757302

[48] Koutsiaris AG, Tachmitzi SV, Batis N, Kotoula MG, Karabatsas CH, Tsironi E, Chatzoulis DZ. Volume flow and wall shear stress quantification in the human conjunctival capillaries and post-capillary venules in vivo. Biorheology. 2007;44(5–6):375–86. 18401076

[49] Ballermann BJ, Dardik A, Eng E, Liu A. Shear stress and the endothelium. Kidney Int. 1998;54:S100–8. 10.1046/j.1523-1755.1998.06720.x 9736263

[50] Abaci HE, Shen Y-I, Tan S, Gerecht S. Recapitulating physiological and pathological shear stress and oxygen to model vasculature in health and disease. Sci Rep. 2014;4(1):4951. 24818558 10.1038/srep04951 PMC4018609

[51] Cheng H, Chen X, Zhong J, Li J, Qiu P, Wang K. Label-free measurement of wall shear stress in the brain venule and arteriole using dual-wavelength third-harmonic-generation line-scanning imaging. Opt Lett. 2022;47(21):5618–21. 37219285 10.1364/OL.472136

[52] Grifno GN, Farrell AM, Linville RM, Arevalo D, Kim JH, Gu L, Searson PC. Tissue-engineered blood-brain barrier models via directed differentiation of human induced pluripotent stem cells. Sci Rep. 2019;9(1):13957. 31562392 10.1038/s41598-019-50193-1 PMC6764995

[53] Candiello J, Cole GJ, Halfter W. Age-dependent changes in the structure, composition and biophysical properties of a human basement membrane. Matrix Biol. 2010;29(5):402–10. 20362054 10.1016/j.matbio.2010.03.004

[54] Gordon E, Schimmel L, Frye M. The importance of mechanical forces for in vitro endothelial cell biology. Front Physiol. 2020;11:684. 32625119 10.3389/fphys.2020.00684 PMC7314997

[55] Kohn JC, Lampi MC, Reinhart-King CA. Age-related vascular stiffening: causes and consequences. Front Genet. 2015. 10.3389/fgene.2015.00112/full. 25926844 10.3389/fgene.2015.00112 PMC4396535

[56] Xue C, Zhang T, Xie X, Zhang Q, Zhang S, Zhu B, et al. Substrate stiffness regulates arterial-venous differentiation of endothelial progenitor cells via the Ras/Mek pathway. Biochim Biophys Acta Mol Cell Res. 2017;1864(10):1799–808. 28732675 10.1016/j.bbamcr.2017.07.006

[57] Marshall BT, Long M, Piper JW, Yago T, McEver RP, Zhu C. Direct observation of catch bonds involving cell-adhesion molecules. Nature. 2003;423(6936):190–3. 12736689 10.1038/nature01605

[58] Huse M. Mechanical forces in the immune system. Nat Rev Immunol. 2017;17(11):679–90. 28757604 10.1038/nri.2017.74 PMC6312705

[59] Zhu C, McEver RP. Catch bonds: physical models and biological functions. Mol Cell Biomech. 2005;2(3):91. 16708472

[60] Morikis VA, Chase S, Wun T, Chaikof EL, Magnani JL, Simon SI. Selectin catch-bonds mechanotransduce integrin activation and neutrophil arrest on inflamed endothelium under shear flow. Blood. 2017;130(19):2101–10. 28811304 10.1182/blood-2017-05-783027 PMC5680610

[61] Kong F, García AJ, Mould AP, Humphries MJ, Zhu C. Demonstration of catch bonds between an integrin and its ligand. J Cell Biol. 2009;185(7):1275–84. 19564406 10.1083/jcb.200810002 PMC2712956

[62] Chen W, Lou J, Zhu C. Forcing switch from short- to intermediate- and long-lived states of the αa domain generates LFA-1/ICAM-1 catch bonds. J Biol Chem. 2010;285(46):35967–78. 20819952 10.1074/jbc.M110.155770 PMC2975219

[63] Valignat MP, Theodoly O, Gucciardi A, Hogg N, Lellouch AC. T lymphocytes orient against the direction of fluid flow during LFA-1-mediated migration. Biophys J. 2013;104(2):322–31. 23442854 10.1016/j.bpj.2012.12.007 PMC3552278

[64] Baumgartner W, Hinterdorfer P, Ness W, Raab A, Vestweber D, Schindler H, Drenckhahn D. Cadherin interaction probed by atomic force microscopy. Proc Natl Acad Sci USA. 2000;97(8):4005–10. 10759550 10.1073/pnas.070052697 PMC18132

[65] Arslan FN, Eckert J, Schmidt T, Heisenberg CP. Holding it together: when cadherin meets cadherin. Biophys J. 2021;120(19):4182–92. 33794149 10.1016/j.bpj.2021.03.025 PMC8516678

[66] Roux E, Bougaran P, Dufourcq P, Couffinhal T. Fluid shear stress sensing by the endothelial layer. Front Physiol. 2020. 10.3389/fphys.2020.00861. 32848833 10.3389/fphys.2020.00861 PMC7396610

[67] Dewey CF Jr, Bussolari SR, Gimbrone MA Jr, Davies PF. The dynamic response of vascular endothelial cells to fluid shear stress. J Biomech Eng. 1981;103(3):177–85. 7278196 10.1115/1.3138276

[68] Rochfort KD, Cummins PM. In vitro cell models of the human blood-brain barrier: demonstrating the beneficial influence of shear stress on brain microvascular endothelial cell phenotype. In: Barichello T, editor. Blood-brain barrier. New York: Springer; 2019. p. 71–98.

[69] Krizanac-Bengez L, Mayberg MR, Janigro D. The cerebral vasculature as a therapeutic target for neurological disorders and the role of shear stress in vascular homeostatis and pathophysiology. Neurol Res. 2004;26(8):846–53. 15727268 10.1179/016164104X3789

[70] Liu Z, Ling SD, Liang K, Chen Y, Niu Y, Sun L, et al. Viscoelasticity of ECM and cells—origin, measurement and correlation. Mechanobiol Med. 2024;2(4):100082. 10.1016/j.mbm.2024.100082 PMC12082326 40395221

[71] Dessalles CA, Leclech C, Castagnino A, Barakat AI. Integration of substrate- and flow-derived stresses in endothelial cell mechanobiology. Commun Biol. 2021;4(1):764. 34155305 10.1038/s42003-021-02285-w PMC8217569

[72] Tran M, Heo C, Lee LP, Cho H. Human mini-blood–brain barrier models for biomedical neuroscience research: a review. Biomater Res. 2022;26(1):82. 36527159 10.1186/s40824-022-00332-z PMC9756735

[73] Kim J, Shin S-A, Lee CS, Chung HJ. An improved in vitro blood-brain barrier model for the evaluation of drug permeability using transwell with shear stress. Pharmaceutics. 2024;16(1):48. 10.3390/pharmaceutics16010048 PMC10820479 38258059

[74] Molins Gutiérrez G, Martorell J, Salazar-Martin AG, Balcells M. A dynamic, in vitro BBB model to study the effects of varying levels of shear stress. In: Stone N, editor. The blood-brain barrier: methods and protocols. New York: Springer; 2022. p. 175–90. 10.1007/978-1-0716-2289-6_10 35733045

[75] Korte N, Nortley R, Attwell D. Cerebral blood flow decrease as an early pathological mechanism in Alzheimer’s disease. Acta Neuropathol. 2020;140(6):793–810. 32865691 10.1007/s00401-020-02215-w PMC7666276

[76] Baeyens N, Nicoli S, Schwartz MA. Vascular remodeling is governed by a VEGFR3-dependent fluid shear stress set point. Elife. 2015;4:e04645. 25643397 10.7554/eLife.04645 PMC4337723

[77] Garcia-Polite F, Martorell J, Del Rey-Puech P, Melgar-Lesmes P, O’Brien CC, Roquer J, et al. Pulsatility and high shear stress deteriorate barrier phenotype in brain microvascular endothelium. J Cereb Blood Flow Metab. 2017;37(7):2614–25. 27702879 10.1177/0271678X16672482 PMC5531355

[78] Swain SM, Liddle RA. Piezo1 acts upstream of TRPV4 to induce pathological changes in endothelial cells due to shear stress. J Biol Chem. 2021;296:100171. 33298523 10.1074/jbc.RA120.015059 PMC7948745

[79] Pasi M, Cordonnier C. Clinical relevance of cerebral small vessel diseases. Stroke. 2020;51(1):47–53. 31752613 10.1161/STROKEAHA.119.024148

[80] Pasi M, Charidimou A, Boulouis G, Auriel E, Ayres A, Schwab KM, et al. Mixed-location cerebral hemorrhage/microbleeds: Underlying microangiopathy and recurrence risk. Neurology. 2018;90(2):e119–26. 29247070 10.1212/WNL.0000000000004797 PMC5772153

[81] Goeldlin MB, Mueller M, Siepen BM, Zhang W, Ozkan H, Locatelli M, et al. CADMUS. Neurology. 2024;102(1):e207977. 38165372 10.1212/WNL.0000000000207977 PMC10834115

[82] Joutel A, Faraci FM. Cerebral small vessel disease. Stroke. 2014;45(4):1215–21. 24503668 10.1161/STROKEAHA.113.002878 PMC3966958

[83] Wartolowska KA, Webb AJS. Midlife blood pressure is associated with the severity of white matter hyperintensities: analysis of the UK biobank cohort study. Eur Heart J. 2021;42(7):750–7. 33238300 10.1093/eurheartj/ehaa756 PMC7882359

[84] Bartstra JW, van den Beukel T, Kranenburg G, Geurts LJ, den Harder AM, Witkamp T, et al. Increased intracranial arterial pulsatility and microvascular brain damage in pseudoxanthoma elasticum. Am J Neuroradiol. 2024;45(4):386–92. 38548304 10.3174/ajnr.A8212 PMC11288551

[85] Lu W, Yu C, Wang L, Wang F, Qiu J. Perfusion heterogeneity of cerebral small vessel disease revealed via arterial spin labeling MRI and machine learning. Neuroimage Clin. 2022;36:103165. 36037662 10.1016/j.nicl.2022.103165 PMC9434130

[86] Jiang X, Andjelkovic AV, Zhu L, Yang T, Bennett MVL, Chen J, et al. Blood-brain barrier dysfunction and recovery after ischemic stroke. Prog Neurobiol. 2018;163–164:144–71. 28987927 10.1016/j.pneurobio.2017.10.001 PMC5886838

[87] Chang E, O’Donnell ME, Barakat AI. Shear stress and 17beta-estradiol modulate cerebral microvascular endothelial Na-K-Cl cotransporter and Na/H exchanger protein levels. Am J Physiol Cell Physiol. 2008;294(1):C363–71. 17959724 10.1152/ajpcell.00045.2007

[88] Zhang M, Liu Q, Meng H, Duan H, Liu X, Wu J, et al. Ischemia-reperfusion injury: molecular mechanisms and therapeutic targets. Signal Transduct Target Ther. 2024;9(1):12. 38185705 10.1038/s41392-023-01688-x PMC10772178

[89] Ashby JW, Mack JJ. Endothelial control of cerebral blood flow. Am J Pathol. 2021;191(11):1906–16. 33713686 10.1016/j.ajpath.2021.02.023

[90] Chen D, Li L, Wang Y, Xu R, Peng S, Zhou L, Deng Z. Ischemia-reperfusion injury of brain induces endothelial-mesenchymal transition and vascular fibrosis via activating let-7i/TGF-βR1 double-negative feedback loop. FASEB J. 2020;34(5):7178–91. 32274860 10.1096/fj.202000201R

[91] Sun D, Ma J, Du L, Liu Q, Yue H, Peng C, et al. Fluid shear stress induced-endothelial phenotypic transition contributes to cerebral ischemia–reperfusion injury and repair. APL Bioeng. 2024. 10.1063/5.0174825. 38414635 10.1063/5.0174825 PMC10898918

[92] Protzmann J, Jung F, Jakobsson L, Fredriksson L. Analysis of ischemic stroke-mediated effects on blood–brain barrier properties along the arteriovenous axis assessed by intravital two-photon imaging. Fluids Barriers CNS. 2024;21(1):35. 38622710 10.1186/s12987-024-00537-5 PMC11017501

[93] Latour LL, Kang DW, Ezzeddine MA, Chalela JA, Warach S. Early blood-brain barrier disruption in human focal brain ischemia. Ann Neurol. 2004;56(4):468–77. 15389899 10.1002/ana.20199

[94] Schuff N, Matsumoto S, Kmiecik J, Studholme C, Du A, Ezekiel F, et al. Cerebral blood flow in ischemic vascular dementia and Alzheimer’s disease, measured by arterial spin-labeling magnetic resonance imaging. Alzheimers Dement. 2009;5(6):454–62. 19896584 10.1016/j.jalz.2009.04.1233 PMC2802181

[95] Tomoto T, Tarumi T, Zhang R. Central arterial stiffness, brain white matter hyperintensity and total brain volume across the adult lifespan. J Hypertens. 2023. 10.1097/HJH.0000000000003404. 36883450 10.1097/HJH.0000000000003404 PMC10079586

[96] Tarumi T, Zhang R. Cerebral blood flow in normal aging adults: cardiovascular determinants, clinical implications, and aerobic fitness. J Neurochem. 2018;144(5):595–608. 28986925 10.1111/jnc.14234 PMC5874160

[97] Law M, Saindane AM, Ge Y, Babb JS, Johnson G, Mannon LJ, et al. Microvascular abnormality in relapsing-remitting multiple sclerosis: perfusion mr imaging findings in normal-appearing white matter. Radiology. 2004;231(3):645–52. 15163806 10.1148/radiol.2313030996

[98] Adhya S, Johnson G, Herbert J, Jaggi H, Babb JS, Grossman RI, Inglese M. Pattern of hemodynamic impairment in multiple sclerosis: dynamic susceptibility contrast perfusion MR imaging at 3.0 T. Neuroimage. 2006;33(4):1029–35. 16996280 10.1016/j.neuroimage.2006.08.008 PMC1752216

[99] Senzaki K, Okada Y, Ochi H, Ochi M, Takei SI, Miura S, et al. Vascular endothelial dysfunction associated with severity in multiple sclerosis. Multiple Scler Related Disorders. 2021. 10.1016/j.msard.2021.103135. 10.1016/j.msard.2021.103135 34274738

[100] Mokhber N, Shariatzadeh A, Avan A, Saber H, Babaei GS, Chaimowitz G, Azarpazhooh MR. Cerebral blood flow changes during aging process and in cognitive disorders: a review. Neuroradiol J. 2021;34(4):300–7. 33749402 10.1177/19714009211002778 PMC8447819

[101] Sankar SB, Pybus AF, Liew A, Sanders B, Shah KJ, Wood LB, Buckley EM. Low cerebral blood flow is a non-invasive biomarker of neuroinflammation after repetitive mild traumatic brain injury. Neurobiol Dis. 2019;124:544–54. 30592976 10.1016/j.nbd.2018.12.018 PMC6528169

[102] Chen Y, Shen F, Liu J, Yang GY. Arterial stiffness and stroke: de-stiffening strategy, a therapeutic target for stroke. Stroke Vasc Neurol. 2017;2(2):65–72. 28959494 10.1136/svn-2016-000045 PMC5600012

[103] Song J, Zhang X, Buscher K, Wang Y, Wang H, Di Russo J, et al. Endothelial basement membrane laminin 511 contributes to endothelial junctional tightness and thereby inhibits leukocyte transmigration. Cell Rep. 2017;18(5):1256–69. 28147279 10.1016/j.celrep.2016.12.092

[104] Thomsen MS, Routhe LJ, Moos T. The vascular basement membrane in the healthy and pathological brain. J Cereb Blood Flow Metab. 2017;37(10):3300–17. 28753105 10.1177/0271678X17722436 PMC5624399

[105] Yao Y. Basement membrane and stroke. J Cereb Blood Flow Metab. 2019;39(1):3–19. 30226080 10.1177/0271678X18801467 PMC6311666

[106] Kretschmer M, Rüdiger D, Zahler S. Mechanical aspects of angiogenesis. Cancers. 2021. 10.3390/cancers13194987. 34638470 10.3390/cancers13194987 PMC8508205

[107] Baeten KM, Akassoglou K. Extracellular matrix and matrix receptors in blood-brain barrier formation and stroke. Dev Neurobiol. 2011;71(11):1018–39. 21780303 10.1002/dneu.20954 PMC3482610

[108] Aman J, Margadant C. Integrin-dependent cell-matrix adhesion in endothelial health and disease. Circ Res. 2023;132(3):355–78. 36730379 10.1161/CIRCRESAHA.122.322332 PMC9891302

[109] Nguyen B, Bix G, Yao Y. Basal lamina changes in neurodegenerative disorders. Mol Neurodegener. 2021;16(1):81. 34876200 10.1186/s13024-021-00502-y PMC8650282

[110] Osada T, Gu YH, Kanazawa M, Tsubota Y, Hawkins BT, Spatz M, et al. Interendothelial claudin-5 expression depends on cerebral endothelial cell-matrix adhesion by β(1)-integrins. J Cereb Blood Flow Metab. 2011;31(10):1972–85. 21772312 10.1038/jcbfm.2011.99 PMC3208159

[111] Kohn JC, Zhou DW, Bordeleau F, Zhou A, Mason B, Mitchell M, et al. Cooperative effects of matrix stiffness and fluid shear stress on endothelial cell behavior. Biophys J. 2015;108(3):471–8. 25650915 10.1016/j.bpj.2014.12.023 PMC4317546

[112] Xue C, Zhang T, Xie X, Zhang Q, Zhang S, Zhu B, et al. Substrate stiffness regulates arterial-venous differentiation of endothelial progenitor cells via the Ras/Mek pathway. Biochim et Biophys Acta (BBA) - Mol Cell Res. 2017;1864(10):1799–808. 10.1016/j.bbamcr.2017.07.006 28732675

[113] Birukov KG, Birukova AA, Dudek SM, Verin AD, Crow MT, Zhan X, et al. Shear stress-mediated cytoskeletal remodeling and cortactin translocation in pulmonary endothelial cells. Am J Respir Cell Mol Biol. 2002;26(4):453–64. 11919082 10.1165/ajrcmb.26.4.4725

[114] Uspenskaia O, Liebetrau M, Herms J, Danek A, Hamann GF. Aging is associated with increased collagen type IV accumulation in the basal lamina of human cerebral microvessels. BMC Neurosci. 2004;5:37. 15387892 10.1186/1471-2202-5-37 PMC523851

[115] Hawkes CA, Gatherer M, Sharp MM, Dorr A, Yuen HM, Kalaria R, et al. Regional differences in the morphological and functional effects of aging on cerebral basement membranes and perivascular drainage of amyloid-β from the mouse brain. Aging Cell. 2013;12(2):224–36. 23413811 10.1111/acel.12045

[116] Reed MJ, Damodarasamy M, Banks WA. The extracellular matrix of the blood-brain barrier: structural and functional roles in health, aging, and Alzheimer’s disease. Tissue Barriers. 2019;7(4):1651157. 31505997 10.1080/21688370.2019.1651157 PMC6866683

[117] Farkas E, De Jong GI, de Vos RAI, Jansen Steur ENH, Luiten PGM. Pathological features of cerebral cortical capillaries are doubled in Alzheimer’s disease and Parkinson’s disease. Acta Neuropathol. 2000;100(4):395–402. 10985698 10.1007/s004010000195

[118] Mancardi GL, Perdelli F, Rivano C, Leonardi A, Bugiani O. Thickening of the basement membrane of cortical capillaries in Alzheimer’s disease. Acta Neuropathol. 1980;49(1):79–83. 7355675 10.1007/BF00692225

[119] Heinz A. Elastic fibers during aging and disease. Ageing Res Rev. 2021;66:101255. 33434682 10.1016/j.arr.2021.101255

[120] Fonck E, Feigl GG, Fasel J, Sage D, Unser M, Rüfenacht DA, Stergiopulos N. Effect of aging on elastin functionality in human cerebral arteries. Stroke. 2009;40(7):2552–6. 19478233 10.1161/STROKEAHA.108.528091

[121] Girouard H, Iadecola C. Neurovascular coupling in the normal brain and in hypertension, stroke, and Alzheimer disease. J Appl Physiol. 2006;100(1):328–35. 16357086 10.1152/japplphysiol.00966.2005

[122] Mandalà M, Cipolla MJ. Aging-related structural and functional changes in cerebral arteries: caloric restriction (CR) intervention. J Vasc Med Surg. 2021. 10.1126/science.aav9518. 34981030 PMC8720434

[123] Nortley R, Korte N, Izquierdo P, Hirunpattarasilp C, Mishra A, Jaunmuktane Z, et al. Amyloid β oligomers constrict human capillaries in Alzheimer’s disease via signaling to pericytes. Science. 2019. 10.1126/science.aav9518. 31221773 10.1126/science.aav9518. PMC6658218

[124] Gürler G, Soylu KO, Yemisci M. Importance of pericytes in the pathophysiology of cerebral ischemia. Noro Psikiyatr Ars. 2022;59(Suppl 1):S29-s35. 36578988 10.29399/npa.28171 PMC9767130

[125] Verzijl N, DeGroot J, Thorpe SR, Bank RA, Shaw JN, Lyons TJ, et al. Effect of collagen turnover on the accumulation of advanced glycation end products*. J Biol Chem. 2000;275(50):39027–31. 10976109 10.1074/jbc.M006700200

[126] Singh J, Di Ferrante N, Gyorkey F, Wilson N. Plasma glycosaminoglycans in normal individuals of various age. Atherosclerosis. 1977;28(3):319–24. 597344 10.1016/0021-9150(77)90179-4

[127] Libby JR, Royce H, Walker SR, Li L. The role of extracellular matrix in angiogenesis: beyond adhesion and structure. Biomater Biosyst. 2024;15:100097. 39129826 10.1016/j.bbiosy.2024.100097 PMC11315062

[128] Lepelletier FX, Mann D, Robinson A, Pinteaux E, Boutin H. Early changes in extracellular matrix in Alzheimer’s disease. Neuropathol Appl Neurobiol. 2017;43(2):167–82. 26544797 10.1111/nan.12295

[129] Tian J, Shi J, Smallman R, Iwatsubo T, Mann DMA. Relationships in Alzheimer’s disease between the extent of Aβ deposition in cerebral blood vessel walls, as cerebral amyloid angiopathy, and the amount of cerebrovascular smooth muscle cells and collagen. Neuropathol Appl Neurobiol. 2006;32(3):332–40. 16640651 10.1111/j.1365-2990.2006.00732.x

[130] Benedict RH, DeLuca J, Phillips G, LaRocca N, Hudson LD, Rudick R. Validity of the symbol digit modalities test as a cognition performance outcome measure for multiple sclerosis. Mult Scler. 2017;23(5):721–33. 28206827 10.1177/1352458517690821 PMC5405816

[131] Zheng P, Pilutti LA, DuBose NG, Motl RW. Vascular function and cognition in persons with multiple sclerosis: preliminary examination. Multiple Scler Related Disorders. 2023;71:104578. 10.1016/j.msard.2023.104578 36805173

[132] Bae JH, Kim JM, Park KY, Han SH. Association between arterial stiffness and the presence of cerebral small vessel disease markers. Brain Behav. 2021;11(1):e01935. 33211410 10.1002/brb3.1935 PMC7821631

[133] Miyagi T, Ishida A, Shinzato T, Ohya Y. Arterial stiffness is associated with small vessel disease irrespective of blood pressure in stroke-free individuals. Stroke. 2023;54(11):2814–21. 37846566 10.1161/STROKEAHA.123.042512

[134] Boshra H, Awad M, Hussein M, Elyamani E. Vascular dysfunction and dyslipidemia in multiple sclerosis: are they correlated with disease duration and disability status? Egypt Heart J. 2022;74(1):9. 35147792 10.1186/s43044-022-00244-2 PMC8837734

[135] Alvarez-Bueno C, Cunha PG, Martinez-Vizcaino V, Pozuelo-Carrascosa DP, Visier-Alfonso ME, Jimenez-Lopez E, Cavero-Redondo I. Arterial stiffness and cognition among adults: a systematic review and meta-analysis of observational and longitudinal studies. J Am Heart Assoc. 2020;9(5):e014621. 32106748 10.1161/JAHA.119.014621 PMC7335587

[136] Ohashi KL, Tung DK, Wilson J, Zweifach BW, Schmid-Schönbein GW. Transvascular and interstitial migration of neutrophils in rat mesentery. Microcirculation. 1996;3(2):199–210. 8839442 10.3109/10739689609148289

[137] Yadav R, Larbi KY, Young RE, Nourshargh S. Migration of leukocytes through the vessel wall and beyond. Thromb Haemost. 2003;90(4):598–606. 14515179 10.1160/TH03-04-0220

[138] Bartholomäus I, Kawakami N, Odoardi F, Schläger C, Miljkovic D, Ellwart JW, et al. Effector T cell interactions with meningeal vascular structures in nascent autoimmune CNS lesions. Nature. 2009;462(7269):94–8. 19829296 10.1038/nature08478

[139] Zhang X, Wang Y, Song J, Gerwien H, Chuquisana O, Chashchina A, et al. The endothelial basement membrane acts as a checkpoint for entry of pathogenic T cells into the brain. J Exp Med. 2020. 10.1084/jem.20191339. 32379272 10.1084/jem.20191339 PMC7336306

[140] Riddle RB, Jennbacken K, Hansson KM, Harper MT. Endothelial inflammation and neutrophil transmigration are modulated by extracellular matrix composition in an inflammation-on-a-chip model. Sci Rep. 2022;12(1):6855. 35477984 10.1038/s41598-022-10849-x PMC9046410

[141] Mutgan AC, Jandl K, Kwapiszewska G. Endothelial basement membrane components and their products, matrikines: active drivers of pulmonary hypertension? Cells. 2020. 10.3390/cells9092029. 32899187 10.3390/cells9092029 PMC7563239

[142] Leclech C, Natale CF, Barakat AI. The basement membrane as a structured surface—role in vascular health and disease. J Cell Sci. 2020. 10.1242/jcs.239889. 32938688 10.1242/jcs.239889

[143] Engelhardt B. Regulation of immune cell entry into the central nervous system. Results Probl Cell Differ. 2006;43:259–80. 17068976 10.1007/400_020

[144] Haage V, De Jager PL. Neuroimmune contributions to Alzheimer’s disease: a focus on human data. Mol Psychiatry. 2022;27(8):3164–81. 35668160 10.1038/s41380-022-01637-0 PMC9168642

[145] Merlini M, Kirabali T, Kulic L, Nitsch RM, Ferretti MT. Extravascular CD3+ T cells in brains of Alzheimer disease patients correlate with tau but not with amyloid pathology: an immunohistochemical study. Neurodegener Dis. 2018;18(1):49–56. 29402847 10.1159/000486200

[146] Zeng J, Liao Z, Yang H, Wang Q, Wu Z, Hua F, Zhou Z. T cell infiltration mediates neurodegeneration and cognitive decline in Alzheimer’s disease. Neurobiol Dis. 2024;193:106461. 38437992 10.1016/j.nbd.2024.106461

[147] Rossy J, Laufer JM, Legler DF. Role of mechanotransduction and tension in T cell function. Front Immunol. 2018. 10.3389/fimmu.2018.02638. 30519239 10.3389/fimmu.2018.02638 PMC6251326

[148] Schaefer A, Hordijk PL. Cell-stiffness-induced mechanosignaling—a key driver of leukocyte transendothelial migration. J Cell Sci. 2015;128(13):2221–30. 26092932 10.1242/jcs.163055

[149] Sun C, Migliorini C, Munn LL. Red blood cells initiate leukocyte rolling in postcapillary expansions: a lattice boltzmann analysis. Biophys J. 2003;85(1):208–22. 12829477 10.1016/S0006-3495(03)74467-1 PMC1303078

[150] Muller WA. Getting leukocytes to the site of inflammation. Vet Pathol. 2013;50(1):7–22. 23345459 10.1177/0300985812469883 PMC3628536

[151] Zhang X, Chen A, De Leon D, Li H, Noiri E, Moy VT, Goligorsky MS. Atomic force microscopy measurement of leukocyte-endothelial interaction. Am J Physiol Heart Circ Physiol. 2004;286(1):H359–67. 12969892 10.1152/ajpheart.00491.2003

[152] Engelhardt B, Ransohoff RM. Capture, crawl, cross: the T cell code to breach the blood–brain barriers. Trends Immunol. 2012;33(12):579–89. 22926201 10.1016/j.it.2012.07.004

[153] Siegelman MH, Stanescu D, Estess P. The CD44-initiated pathway of T-cell extravasation uses VLA-4 but not LFA-1 for firm adhesion. J Clin Invest. 2000;105(5):683–91. 10712440 10.1172/JCI8692 PMC292454

[154] Coisne C, Faveeuw C, Delplace Y, Dehouck L, Miller F, Cecchelli R, Dehouck B. Differential expression of selectins by mouse brain capillary endothelial cells in vitro in response to distinct inflammatory stimuli. Neurosci Lett. 2006;392(3):216–20. 16214291 10.1016/j.neulet.2005.09.028

[155] McDonnell GV, McMillan SA, Douglas JP, Droogan AG, Hawkins SA. Serum soluble adhesion molecules in multiple sclerosis: raised sVCAM-1, sICAM-1 and sE-selectin in primary progressive disease. J Neurol. 1999;246(2):87–92. 10195402 10.1007/s004150050313

[156] Yousef H, Czupalla CJ, Lee D, Chen MB, Burke AN, Zera KA, et al. Aged blood impairs hippocampal neural precursor activity and activates microglia via brain endothelial cell VCAM1. Nat Med. 2019;25(6):988–1000. 31086348 10.1038/s41591-019-0440-4 PMC6642642

[157] Sathiyanadan K, Coisne C, Enzmann G, Deutsch U, Engelhardt B. PSGL-1 and E/P-selectins are essential for T-cell rolling in inflamed CNS microvessels but dispensable for initiation of EAE. Eur J Immunol. 2014;44(8):2287–94. 24740164 10.1002/eji.201344214

[158] Nishihara H, Soldati S, Mossu A, Rosito M, Rudolph H, Muller WA, et al. Human CD4+ T cell subsets differ in their abilities to cross endothelial and epithelial brain barriers in vitro. Fluids and Barriers of the CNS. 2020;17(1):3. 32008573 10.1186/s12987-019-0165-2 PMC6996191

[159] Mapunda JA, Tibar H, Regragui W, Engelhardt B. How does the immune system enter the brain? Front Immunol. 2022. 10.3389/fimmu.2022.805657/full. 35273596 10.3389/fimmu.2022.805657 PMC8902072

[160] Lyck R, Engelhardt B. Going against the tide—how encephalitogenic T cells breach the blood-brain barrier. J Vasc Res. 2012;49(6):497–509. 22948545 10.1159/000341232

[161] Steiner O, Coisne C, Cecchelli R, Boscacci R, Deutsch U, Engelhardt B, Lyck R. Differential roles for endothelial ICAM-1, ICAM-2, and VCAM-1 in shear-resistant T cell arrest, polarization, and directed crawling on blood-brain barrier endothelium. J Immunol. 2010;185(8):4846–55. 20861356 10.4049/jimmunol.0903732

[162] Zhu C, Chen W, Lou J, Rittase W, Li K. Mechanosensing through immunoreceptors. Nat Immunol. 2019;20(10):1269–78. 31534240 10.1038/s41590-019-0491-1 PMC7592628

[163] Pageon SV, Govendir MA, Kempe D, Biro M. Mechanoimmunology: molecular-scale forces govern immune cell functions. Mol Biol Cell. 2018;29(16):1919–26. 30088799 10.1091/mbc.E18-02-0120 PMC6232972

[164] Liu B, Chen W, Zhu C. Accumulation of dynamic catch bonds between TCR and agonist peptide-MHC triggers T cell signaling. Cell. 2014;157(2):357–68. 24725404 10.1016/j.cell.2014.02.053 PMC4123688

[165] Harrison DL, Fang Y, Huang J. T-cell mechanobiology: force sensation, potentiation, and translation. Front Phys. 2019. 10.3389/fphy.2019.00045/full. 32601597 10.3389/fphy.2019.00045 PMC7323161

[166] Alon R, Dustin ML. Force as a facilitator of integrin conformational changes during leukocyte arrest on blood vessels and antigen-presenting cells. Immunity. 2007;26(1):17–27. 17241958 10.1016/j.immuni.2007.01.002

[167] Sun Z, Costell M, Fässler R. Integrin activation by talin, kindlin and mechanical forces. Nat Cell Biol. 2019;21(1):25–31. 30602766 10.1038/s41556-018-0234-9

[168] Zhu J, Luo B-H, Xiao T, Zhang C, Nishida N, Springer TA. Structure of a complete integrin ectodomain in a physiologic resting state and activation and deactivation by applied forces. Mol Cell. 2008;32(6):849–61. 19111664 10.1016/j.molcel.2008.11.018 PMC2758073

[169] Shen B, Delaney MK, Du X. Inside-out, outside-in, and inside-outside-in: G protein signaling in integrin-mediated cell adhesion, spreading, and retraction. Curr Opin Cell Biol. 2012;24(5):600–6. 22980731 10.1016/j.ceb.2012.08.011 PMC3479359

[170] Shao B, Yago T, Panicker SR, Zhang N, Liu Z, McEver RP. Th1 cells rolling on selectins trigger DAP12-dependent signals that activate integrin αLβ2. J Immunol. 2020;204(1):37–48. 31757864 10.4049/jimmunol.1900680 PMC6920551

[171] Woolf E, Grigorova I, Sagiv A, Grabovsky V, Feigelson SW, Shulman Z, et al. Lymph node chemokines promote sustained T lymphocyte motility without triggering stable integrin adhesiveness in the absence of shear forces. Nat Immunol. 2007;8(10):1076–85. 17721537 10.1038/ni1499

[172] Dominguez GA, Anderson NR, Hammer DA. The direction of migration of T-lymphocytes under flow depends upon which adhesion receptors are engaged. Integr Biol. 2015;7(3):345–55. 10.1039/c4ib00201f PMC4746477 25674729

[173] Lee J, Song KH, Kim T, Doh J. Endothelial cell focal adhesion regulates transendothelial migration and subendothelial crawling of T cells. Front Immunol. 2018. 10.3389/fimmu.2018.00048/full. 29472915 10.3389/fimmu.2018.00048 PMC5810271

[174] Coisne C, Lyck R, Engelhardt B. Live cell imaging techniques to study T cell trafficking across the blood-brain barrier in vitro and in vivo. Fluids Barriers CNS. 2013;10(1):7. 23336847 10.1186/2045-8118-10-7 PMC3560242

[175] Elosegui-Artola A, Oria R, Chen Y, Kosmalska A, Pérez-González C, Castro N, et al. Mechanical regulation of a molecular clutch defines force transmission and transduction in response to matrix rigidity. Nat Cell Biol. 2016;18(5):540–8. 27065098 10.1038/ncb3336

[176] Renkawitz J, Sixt M. Mechanisms of force generation and force transmission during interstitial leukocyte migration. EMBO Rep. 2010;11(10):744–50. 20865016 10.1038/embor.2010.147 PMC2948197

[177] Saitakis M, Dogniaux S, Goudot C, Bufi N, Asnacios S, Maurin M, et al. Different TCR-induced T lymphocyte responses are potentiated by stiffness with variable sensitivity. Elife. 2017. 10.7554/eLife.23190. 28594327 10.7554/eLife.23190 PMC5464771

[178] Schaefer A, Te Riet J, Ritz K, Hoogenboezem M, Anthony EC, Mul FPJ, et al. Actin-binding proteins differentially regulate endothelial cell stiffness, ICAM-1 function and neutrophil transmigration. J Cell Sci. 2014;127(20):4470–82. 25107367 10.1242/jcs.154708

[179] Sumagin R, Prizant H, Lomakina E, Waugh RE, Sarelius IH. LFA-1 and Mac-1 define characteristically different intralumenal crawling and emigration patterns for monocytes and neutrophils in situ. J Immunol. 2010;185(11):7057–66. 21037096 10.4049/jimmunol.1001638 PMC3004223

[180] Kim SHJ, Hammer DA. Integrin crosstalk allows CD4+ T lymphocytes to continue migrating in the upstream direction after flow. Integr Biol. 2019;11(10):384–93. 10.1093/intbio/zyz034 PMC6946828 31851360

[181] Schimmel L, Heemskerk N, van Buul JD. Leukocyte transendothelial migration: a local affair. Small GTPases. 2017;8(1):1–15. 27715453 10.1080/21541248.2016.1197872 PMC5331897

[182] Martinelli R, Zeiger A, Whitfield M, Sciuto T, Dvorak A, Van Vliet K, et al. Probing the biomechanical contribution of the endothelium to lymphocyte migration: diapedesis by the path of least resistance. J Cell Sci. 2014. 10.1242/jcs.148619. 25002404 10.1242/jcs.148619 PMC4150060

[183] Abadier M, Haghayegh Jahromi N, Cardoso Alves L, Boscacci R, Vestweber D, Barnum S, et al. Cell surface levels of endothelial ICAM-1 influence the transcellular or paracellular T-cell diapedesis across the blood-brain barrier. Eur J Immunol. 2015;45(4):1043–58. 25545837 10.1002/eji.201445125

[184] Marchetti L, Francisco D, Soldati S, Haghayegh Jahromi N, Barcos S, Gruber I, et al. ACKR1 favors transcellular over paracellular T-cell diapedesis across the blood-brain barrier in neuroinflammation in vitro. Eur J Immunol. 2022;52(1):161–77. 34524684 10.1002/eji.202149238 PMC9293480

[185] Lutz SE, Smith JR, Kim DH, Olson CVL, Ellefsen K, Bates JM, et al. Caveolin1 is required for Th1 cell infiltration, but not tight junction remodeling, at the blood-brain barrier in autoimmune neuroinflammation. Cell Rep. 2017;21(8):2104–17. 29166603 10.1016/j.celrep.2017.10.094 PMC5728697

[186] Trevino TN, Almousawi AA, Ochoa-Raya A, Zemanski K, Oliveira SD, Marottoli FM, et al. Endothelial caveolin-1 and CXCL10 promote transcellular migration of autoreactive T cells across the blood-brain barrier. bioRxiv. 2022. 10.1101/2022.11.15.516689v1.abstract.

[187] Bennett J, Basivireddy J, Kollar A, Biron KE, Reickmann P, Jefferies WA, McQuaid S. Blood–brain barrier disruption and enhanced vascular permeability in the multiple sclerosis model EAE. J Neuroimmunol. 2010;229(1):180–91. 20832870 10.1016/j.jneuroim.2010.08.011

[188] Carman CV. Mechanisms for transcellular diapedesis: probing and pathfinding by `invadosome-like protrusions’. J Cell Sci. 2009;122(17):3025–35. 19692589 10.1242/jcs.047522

[189] Alon R, Van Buul JD. leukocyte breaching of endothelial barriers: the actin link. Trends Immunol. 2017;38(8):606–15. 28559148 10.1016/j.it.2017.05.002

[190] Carman CV, Sage PT, Sciuto TE, De La Fuente MA, Geha RS, et al. Transcellular diapedesis is initiated by invasive podosomes. Immunity. 2007;26(6):784–97. 17570692 10.1016/j.immuni.2007.04.015 PMC2094044

[191] Heemskerk N, Schimmel L, Oort C, Van Rijssel J, Yin T, Ma B, et al. F-actin-rich contractile endothelial pores prevent vascular leakage during leukocyte diapedesis through local RhoA signalling. Nat Commun. 2016;7(1):10493. 26814335 10.1038/ncomms10493 PMC4737874

[192] Smyth LA, Afzali B, Tsang J, Lombardi G, Lechler RI. Intercellular transfer of MHC and immunological molecules: molecular mechanisms and biological significance. Am J Transplant. 2007;7(6):1442–9. 17511673 10.1111/j.1600-6143.2007.01816.x PMC3815510

[193] Brezinschek RI, Oppenheimer-Marks N, Lipsky PE. Activated T cells acquire endothelial cell surface determinants during transendothelial migration. J Immunol. 1999;162(3):1677–84. 9973429

[194] Grönloh MLB, Arts JJG, Palacios Martínez S, van der Veen AA, Kempers L, van Steen ACI, et al. Endothelial transmigration hotspots limit vascular leakage through heterogeneous expression of ICAM-1. EMBO Rep. 2023;24(1):e55483. 36382783 10.15252/embr.202255483 PMC9827561

[195] Dorland YL, Huveneers S. Cell–cell junctional mechanotransduction in endothelial remodeling. Cell Mol Life Sci. 2017;74(2):279–92. 27506620 10.1007/s00018-016-2325-8 PMC5219012

[196] Twiss F, Le Duc Q, Van Der Horst S, Tabdili H, Van Der Krogt G, Wang N, et al. Vinculin-dependent cadherin mechanosensing regulates efficient epithelial barrier formation. Biol Open. 2012;1(11):1128–40. 23213393 10.1242/bio.20122428 PMC3507192

[197] Malinova TS, Angulo-Urarte A, Nüchel J, Tauber M, Van Der Stoel MM, Janssen V, et al. A junctional PACSIN2/EHD4/MICAL-L1 complex coordinates VE-cadherin trafficking for endothelial migration and angiogenesis. Nature Communications. 2021;12(1). 10.1038/s41467-021-22873-y PMC8110786 33972531

[198] Arbore C, Sergides M, Gardini L, Bianchi G, Kashchuk AV, Pertici I, et al. α-catenin switches between a slip and an asymmetric catch bond with F-actin to cooperatively regulate cell junction fluidity. bioRxiv. 2021. 10.1038/s41467-022-28779-7. 10.1038/s41467-022-28779-7 PMC8894357 35241656

[199] Huang DL, Bax NA, Buckley CD, Weis WI, Dunn AR. Vinculin forms a directionally asymmetric catch bond with F-actin. Science. 2017;357(6352):703–6. 28818948 10.1126/science.aan2556 PMC5821505

[200] Owen LM, Bax NA, Weis WI, Dunn AR. The C-terminal actin-binding domain of talin forms an asymmetric catch bond with F-actin. Proc Natl Acad Sci USA. 2022;119(10):e2109329119. 35245171 10.1073/pnas.2109329119 PMC8915792

[201] Arbore C, Sergides M, Gardini L, Bianchi G, Kashchuk AV, Pertici I, et al. α-catenin switches between a slip and an asymmetric catch bond with F-actin to cooperatively regulate cell junction fluidity. Nat Commun. 2022;13(1):1146. 35241656 10.1038/s41467-022-28779-7 PMC8894357

[202] Fang Y, Wu D, Birukov KG. Mechanosensing and mechanoregulation of endothelial cell functions. Compr Physiol. 2019;9(2):873–904. 30873580 10.1002/cphy.c180020 PMC6697421

[203] Dembo M, Torney DC, Saxman K, Hammer D, Murray JD. The reaction-limited kinetics of membrane-to-surface adhesion and detachment. Proc R Soc Lond B. 1988;234(1274):55–83. 2901109 10.1098/rspb.1988.0038

[204] Collins C, Guilluy C, Welch C, O’Brien ET, Hahn K, Superfine R, et al. Localized tensional forces on PECAM-1 elicit a global mechanotransduction response via the integrin-RhoA pathway. Curr Biol. 2012;22(22):2087–94. 23084990 10.1016/j.cub.2012.08.051 PMC3681294

[205] Gavard J, Gutkind JS. VE-cadherin and claudin-5: it takes two to tango. Nat Cell Biol. 2008;10(8):883–5. 18670447 10.1038/ncb0808-883 PMC2666287

[206] Wimmer I, Tietz S, Nishihara H, Deutsch U, Sallusto F, Gosselet F, et al. PECAM-1 stabilizes blood-brain barrier integrity and favors paracellular T-cell diapedesis across the blood-brain barrier during neuroinflammation. Front Immunol. 2019. 10.3389/fimmu.2019.00711. 31024547 10.3389/fimmu.2019.00711 PMC6460670

[207] Tzima E, Irani-Tehrani M, Kiosses WB, Dejana E, Schultz DA, Engelhardt B, et al. A mechanosensory complex that mediates the endothelial cell response to fluid shear stress. Nature. 2005;437(7057):426–31. 16163360 10.1038/nature03952

[208] Collins C, Guilluy C, Welch CE, Hahn K, Superfine R, et al. Localized tensional forces on PECAM-1 elicit a global mechanotransduction response via the integrin-RhoA pathway. Curr Biol. 2012;22(22):2087–94. 23084990 10.1016/j.cub.2012.08.051 PMC3681294

[209] Weber EW, Han F, Tauseef M, Birnbaumer L, Mehta D, Muller WA. TRPC6 is the endothelial calcium channel that regulates leukocyte transendothelial migration during the inflammatory response. J Exp Med. 2015;212(11):1883–99. 26392222 10.1084/jem.20150353 PMC4612081

[210] Winneberger J, Schöls S, Lessmann K, Rández-Garbayo J, Bauer AT, Mohamud Yusuf A, et al. Platelet endothelial cell adhesion molecule-1 is a gatekeeper of neutrophil transendothelial migration in ischemic stroke. Brain Behav Immun. 2021;93:277–87. 33388423 10.1016/j.bbi.2020.12.026

[211] Harry BL, Sanders JM, Feaver RE, Lansey M, Deem TL, Zarbock A, et al. Endothelial cell PECAM-1 promotes atherosclerotic lesions in areas of disturbed flow in ApoE-deficient mice. Arterioscler Thromb Vasc Biol. 2008;28(11):2003–8. 18688018 10.1161/ATVBAHA.108.164707 PMC2651147

[212] Haas AJ, Zihni C, Ruppel A, Hartmann C, Ebnet K, Tada M, et al. Interplay between extracellular matrix stiffness and JAM-A regulates mechanical load on ZO-1 and tight junction assembly. Cell Rep. 2020;32(3):107924. 32697990 10.1016/j.celrep.2020.107924 PMC7383227

[213] Chistiakov DA, Orekhov AN, Bobryshev YV. Effects of shear stress on endothelial cells: go with the flow. Acta Physiol. 2017;219(2):382–408. 10.1111/apha.12725 27246807

[214] Tornavaca O, Chia M, Dufton N, Almagro LO, Conway DE, Randi AM, et al. ZO-1 controls endothelial adherens junctions, cell–cell tension, angiogenesis, and barrier formation. J Cell Biol. 2015;208(6):821–38. 25753039 10.1083/jcb.201404140 PMC4362456

[215] Liu Z, Tan JL, Cohen DM, Yang MT, Sniadecki NJ, Ruiz SA, et al. Mechanical tugging force regulates the size of cell-cell junctions. Proc Natl Acad Sci. 2010;107(22):9944–9. 20463286 10.1073/pnas.0914547107 PMC2890446

[216] Yap AS, Duszyc K, Viasnoff V. Mechanosensing and mechanotransduction at cell-cell junctions. Cold Spring Harb Perspect Biol. 2018. 10.1101/cshperspect.a028761. 28778874 10.1101/cshperspect.a028761 PMC6071489

[217] Shihata W, Michell D, Andrews K, Chin-Dusting J. Caveolae: a role in endothelial inflammation and mechanotransduction? Front Physiol. 2016. 10.3389/fphys.2016.00628. 28066261 10.3389/fphys.2016.00628 PMC5168557

[218] Jin P, Jan LY, Jan Y-N. Mechanosensitive ion channels: structural features relevant to mechanotransduction mechanisms. Ann Rev Neurosci. 2020;43:207–29. 32084327 10.1146/annurev-neuro-070918-050509

[219] Arnadóttir J, Chalfie M. Eukaryotic mechanosensitive channels. Annu Rev Biophys. 2010;39:111–37. 20192782 10.1146/annurev.biophys.37.032807.125836

[220] Beedle AEM, Roca-Cusachs P. The reversibility of cellular mechano-activation. Curr Opin Cell Biol. 2023;84:102229. 37633090 10.1016/j.ceb.2023.102229

[221] Sala S, Caillier A, Oakes PW. Principles and regulation of mechanosensing. J Cell Sci. 2024. 10.1242/jcs.261338. 39297391 10.1242/jcs.261338 PMC11423818

[222] Jin Z-G, Ueba H, Tanimoto T, Lungu AO, Frame MD, Berk BC. Ligand-independent activation of vascular endothelial growth factor receptor 2 by fluid shear stress regulates activation of endothelial nitric oxide synthase. Circ Res. 2003;93(4):354–63. 12893742 10.1161/01.RES.0000089257.94002.96

[223] Jong Lee H, Young KG. Shear stress activates Tie2 receptor tyrosine kinase in human endothelial cells. Biochem Biophys Res Commun. 2003;304(2):399–404. 12711329 10.1016/s0006-291x(03)00592-8

[224] Gavard J, Gutkind JS. VEGF controls endothelial-cell permeability by promoting the beta-arrestin-dependent endocytosis of VE-cadherin. Nat Cell Biol. 2006;8(11):1223–34. 17060906 10.1038/ncb1486

[225] Miller B, Sewell-Loftin MK. Mechanoregulation of vascular endothelial growth factor receptor 2 in angiogenesis. Front Cardiovasc Med. 2021;8:804934. 35087885 10.3389/fcvm.2021.804934 PMC8787114

[226] Matsuo E, Okamoto T, Ito A, Kawamoto E, Asanuma K, Wada K, et al. Substrate stiffness modulates endothelial cell function via the YAP-Dll4-Notch1 pathway. Exp Cell Res. 2021;408(1):112835. 34543658 10.1016/j.yexcr.2021.112835

[227] Wang KC, Yeh YT, Nguyen P, Limqueco E, Lopez J, Thorossian S, et al. Flow-dependent YAP/TAZ activities regulate endothelial phenotypes and atherosclerosis. Proc Natl Acad Sci USA. 2016;113(41):11525–30. 27671657 10.1073/pnas.1613121113 PMC5068257

[228] Huang HC, Wang TY, Rousseau J, Mungaray M, Michaud C, Plaisier C, et al. Lesion-specific suppression of YAP/TAZ by biomimetic nanodrug ameliorates atherosclerosis development. bioRxiv. 2023;16:389.

[229] Jia G, Aroor AR, Jia C, Sowers JR. Endothelial cell senescence in aging-related vascular dysfunction. Biochim Biophys Acta Mol Basis Dis. 2019;1865(7):1802–9. 31109450 10.1016/j.bbadis.2018.08.008

[230] Chachisvilis M, Zhang YL, Frangos JA. G protein-coupled receptors sense fluid shear stress in endothelial cells. Proc Natl Acad Sci. 2006;103(42):15463–8. 17030791 10.1073/pnas.0607224103 PMC1622845

[231] Xu J, Mathur J, Vessières E, Hammack S, Nonomura K, Favre J, et al. GPR68 senses flow and is essential for vascular physiology. Cell. 2018;173(3):762-75.e16. 29677517 10.1016/j.cell.2018.03.076 PMC5951615

[232] Iliff AJ, Xu XZS. A mechanosensitive GPCR that detects the bloody force. Cell. 2018;173(3):542–4. 29677505 10.1016/j.cell.2018.04.001 PMC6706248

[233] Ludwig M-G, Vanek M, Guerini D, Gasser JA, Jones CE, Junker U, et al. Proton-sensing G-protein-coupled receptors. Nature. 2003;425(6953):93–8. 12955148 10.1038/nature01905

[234] Wenzel J, Hansen CE, Bettoni C, Vogt MA, Lembrich B, Natsagdorj R, et al. Impaired endothelium-mediated cerebrovascular reactivity promotes anxiety and respiration disorders in mice. Proc Natl Acad Sci. 2020;117(3):1753–61. 31896584 10.1073/pnas.1907467117 PMC6983400

[235] Wang T, Zhou G, He M, Xu Y, Rusyniak WG, Xu Y, et al. GPR68 is a neuroprotective proton receptor in brain ischemia. Stroke. 2020;51(12):3690–700. 33059544 10.1161/STROKEAHA.120.031479 PMC7678672

[236] McGinley MP, Cohen JA. Sphingosine 1-phosphate receptor modulators in multiple sclerosis and other conditions. Lancet. 2021;398(10306):1184–94. 34175020 10.1016/S0140-6736(21)00244-0

[237] Jung B, Obinata H, Galvani S, Mendelson K, Ding B-S, Skoura A, et al. Flow-regulated endothelial S1P receptor-1 signaling sustains vascular development. Dev Cell. 2012;23(3):600–10. 22975328 10.1016/j.devcel.2012.07.015 PMC3443394

[238] Fu Y, Hao J, Zhang N, Ren L, Sun N, Li YJ, et al. Fingolimod for the treatment of intracerebral hemorrhage: a 2-arm proof-of-concept study. JAMA Neurol. 2014;71(9):1092–101. 25003359 10.1001/jamaneurol.2014.1065

[239] Zhu Z, Fu Y, Tian D, Sun N, Han W, Chang G, et al. Combination of the immune modulator fingolimod with alteplase in acute ischemic stroke: a pilot trial. Circulation. 2015;132(12):1104–12. 26202811 10.1161/CIRCULATIONAHA.115.016371 PMC4580515

[240] Nishihara H, Shimizu F, Sano Y, Takeshita Y, Maeda T, Abe M, et al. Fingolimod prevents blood-brain barrier disruption induced by the sera from patients with multiple sclerosis. PLoS ONE. 2015;10(3):e0121488. 25774903 10.1371/journal.pone.0121488 PMC4361641

[241] Cantalupo A, Gargiulo A, Dautaj E, Liu C, Zhang Y, Hla T, Di Lorenzo A. S1PR1 (sphingosine-1-phosphate receptor 1) signaling regulates blood flow and pressure. Hypertension. 2017;70(2):426–34. 28607130 10.1161/HYPERTENSIONAHA.117.09088 PMC5531041

[242] Hughes R, Dalakas MC, Merkies I, Latov N, Léger J-M, Nobile-Orazio E, et al. Oral fingolimod for chronic inflammatory demyelinating polyradiculoneuropathy (FORCIDP Trial): a double-blind, multicentre, randomised controlled trial. Lancet Neurol. 2018;17(8):689–98. 30001923 10.1016/S1474-4422(18)30202-3

[243] Schwab A, Fabian A, Hanley PJ, Stock C. Role of ion channels and transporters in cell migration. Physiol Rev. 2012;92(4):1865–913. 23073633 10.1152/physrev.00018.2011

[244] Tanaka K, Joshi D, Timalsina S, Schwartz MA. Early events in endothelial flow sensing. Cytoskeleton. 2021;78(6):217–31. 33543538 10.1002/cm.21652

[245] Ma X, Liu W. Calcium signaling in brain microvascular endothelial cells and its roles in the function of the blood-brain barrier. NeuroReport. 2019;30(18):1271–7. 31688421 10.1097/WNR.0000000000001357

[246] Brown RC, Wu L, Hicks K, O’Neil RG. Regulation of blood-brain barrier permeability by transient receptor potential type C and type v calcium-permeable channels. Microcirculation. 2008;15(4):359–71. 18464164 10.1080/10739680701762656 PMC3077823

[247] Berrout J, Jin M, O’Neil RG. Critical role of TRPP2 and TRPC1 channels in stretch-induced injury of blood-brain barrier endothelial cells. Brain Res. 2012;1436:1–12. 22192412 10.1016/j.brainres.2011.11.044

[248] Hansen CE, Kamermans A, Mol K, Berve K, Rodriguez-Mogeda C, Fung WK, et al. Inflammation-induced TRPV4 channels exacerbate blood–brain barrier dysfunction in multiple sclerosis. J Neuroinflammation. 2024;21(1):72. 38521959 10.1186/s12974-024-03069-9 PMC10960997

[249] Li J, Hou B, Tumova S, Muraki K, Bruns A, Ludlow MJ, et al. Piezo1 integration of vascular architecture with physiological force. Nature. 2014;515(7526):279–82. 25119035 10.1038/nature13701 PMC4230887

[250] Harraz OF, Klug NR, Senatore AJ, Hill-Eubanks DC, Nelson MT. Piezo1 is a mechanosensor channel in central nervous system capillaries. Circ Res. 2022;130(10):1531–46. 35382561 10.1161/CIRCRESAHA.122.320827 PMC9106929

[251] Kang H, Hong Z, Zhong M, Klomp J, Bayless KJ, Mehta D, et al. Piezo1 mediates angiogenesis through activation of MT1-MMP signaling. Am J Phys Cell Physiol. 2019;316(1):C92–103. 10.1152/ajpcell.00346.2018 PMC6383143 30427721

[252] Friedrich EE, Hong Z, Xiong S, Zhong M, Di A, Rehman J, et al. Endothelial cell Piezo1 mediates pressure-induced lung vascular hyperpermeability via disruption of adherens junctions. Proc Natl Acad Sci USA. 2019;116(26):12980–5. 31186359 10.1073/pnas.1902165116 PMC6600969

[253] Wang Z, Chen J, Babicheva A, Jain PP, Rodriguez M, Ayon RJ, et al. Endothelial upregulation of mechanosensitive channel Piezo1 in pulmonary hypertension. Am J Physiol Cell Physiol. 2021;321(6):C1010–27. 34669509 10.1152/ajpcell.00147.2021 PMC8714987

[254] Yang Y, Wang D, Zhang C, Yang W, Li C, Gao Z, et al. Piezo1 mediates endothelial atherogenic inflammatory responses via regulation of YAP/TAZ activation. Hum Cell. 2021. 10.1007/s13577-021-00600-5. 34606042 10.1007/s13577-021-00600-5

[255] Albarrán-Juárez J, Iring A, Wang S, Joseph S, Grimm M, Strilic B, et al. Piezo1 and Gq/G11 promote endothelial inflammation depending on flow pattern and integrin activation. J Exp Med. 2018;215(10):2655–72. 30194266 10.1084/jem.20180483 PMC6170174

[256] Chuntharpursat-Bon E, Povstyan OV, Ludlow MJ, Carrier DJ, Debant M, Shi J, et al. PIEZO1 and PECAM1 interact at cell-cell junctions and partner in endothelial force sensing. Commun Biol. 2023;6(1):358. 37005489 10.1038/s42003-023-04706-4 PMC10067937

[257] Bertin S, Raz E. Transient receptor potential (TRP) channels in T cells. Semin Immunopathol. 2016;38(3):309–19. 26468011 10.1007/s00281-015-0535-z PMC4833713

[258] Rosenbaum T, Islas LD. Molecular physiology of TRPV channels: controversies and future challenges. Annu Rev Physiol. 2023;85:293–316. 36763971 10.1146/annurev-physiol-030222-012349

[259] Nikolaev YA, Cox CD, Ridone P, Rohde PR, Cordero-Morales JF, Vásquez V, et al. Mammalian TRP ion channels are insensitive to membrane stretch. J Cell Sci. 2019;132(23):238360. 10.1242/jcs.238360 PMC6918743 31722978

[260] Hill-Eubanks DC, Gonzales AL, Sonkusare SK, Nelson MT. Vascular TRP channels: performing under pressure and going with the flow. Physiology. 2014;29(5):343–60. 25180264 10.1152/physiol.00009.2014 PMC4214829

[261] Dragovich MA, Chester D, Fu BM, Wu C, Xu Y, Goligorsky MS, Zhang XF. Mechanotransduction of the endothelial glycocalyx mediates nitric oxide production through activation of TRP channels. Am J Physiol Cell Physiol. 2016;311(6):C846–53. 27681180 10.1152/ajpcell.00288.2015

[262] Earley S, Gonzales AL, Crnich R. Endothelium-dependent cerebral artery dilation mediated by TRPA1 and Ca 2+ -activated K + channels. Circ Res. 2009;104(8):987–94. 19299646 10.1161/CIRCRESAHA.108.189530 PMC2966339

[263] Tiwari A, Acharya T, Saha S, et al. Transient receptor potential ankyrin1 channel is endogenously expressed in T cells and is involved in immune functions. Biosci Rep. 2019;39(9):20191437. 10.1042/BSR20191437 PMC6753326 31488616

[264] Naert R, López-Requena A, Talavera K. TRPA1 expression and pathophysiology in immune cells. Int J Mol Sci. 2021;22(21):11460. 34768891 10.3390/ijms222111460 PMC8583806

[265] Wenning AS, Neblung K, Strauß B, Wolfs M-J, Sappok A, Hoth M, Schwarz EC. TRP expression pattern and the functional importance of TRPC3 in primary human T-cells. Biochim et Biophys Acta (BBA) - Mol Cell Res. 2011;1813(3):412–23. 10.1016/j.bbamcr.2010.12.022 21215279

[266] Yip H, Chan W-Y, Leung P-C, Kwan H-Y, Liu C, Huang Y, et al. Expression of TRPC homologs in endothelial cells and smooth muscle layers of human arteries. Histochem Cell Biol. 2004;122(6):553–61. 15538613 10.1007/s00418-004-0720-y

[267] Lindemann O, Strodthoff C, Horstmann M, Nielsen N, Jung F, Schimmelpfennig S, et al. TRPC1 regulates fMLP-stimulated migration and chemotaxis of neutrophil granulocytes. Biochim et Biophys Acta (BBA) - Mol Cell Res. 2015;1853(9):2122–30. 10.1016/j.bbamcr.2014.12.037 25595528

[268] Chauhan A, Sun Y, Sukumaran P, Quenum Zangbede FO, Jondle CN, Sharma A, et al. M1 macrophage polarization is dependent on TRPC1-mediated calcium entry. iScience. 2018;8:85–102. 30293012 10.1016/j.isci.2018.09.014 PMC6174824

[269] Bréchard S, Melchior C, Plançon S, Schenten V, Tschirhart EJ. Store-operated Ca2+ channels formed by TRPC1, TRPC6 and orai1 and non-store-operated channels formed by TRPC3 are involved in the regulation of NADPH oxidase in HL-60 granulocytes. Cell Calcium. 2008;44(5):492–506. 18436303 10.1016/j.ceca.2008.03.002

[270] Tano JY, Solanki S, Lee RH, Smedlund K, Birnbaumer L, Vazquez G. Bone marrow deficiency of TRPC3 channel reduces early lesion burden and necrotic core of advanced plaques in a mouse model of atherosclerosis. Cardiovasc Res. 2014;101(1):138–44. 24101197 10.1093/cvr/cvt231 PMC3868349

[271] Numaga T, Nishida M, Kiyonaka S, Kato K, Katano M, Mori E, et al. Ca2+ influx and protein scaffolding via TRPC3 sustain PKCbeta and ERK activation in B cells. J Cell Sci. 2010;123(Pt 6):927–38. 20179100 10.1242/jcs.061051 PMC2831761

[272] Quick K, Zhao J, Eijkelkamp N, Linley JE, Rugiero F, Cox JJ, et al. TRPC3 and TRPC6 are essential for normal mechanotransduction in subsets of sensory neurons and cochlear hair cells. Open Biol. 2012;2(5):120068. 22724068 10.1098/rsob.120068 PMC3376737

[273] Tao L, Guo G, Qi Y, Xiong Y, Ma X, Wu N, et al. Inhibition of canonical transient receptor potential 5 channels polarizes macrophages to an M1 phenotype. Pharmacology. 2020;105(3–4):202–8. 31618743 10.1159/000503452

[274] Shen B, Wong C-O, Lau O-C, Woo T, Bai S, Huang Y, Yao X. Plasma membrane mechanical stress activates TRPC5 channels. PLoS ONE. 2015;10(4):e0122227. 25849346 10.1371/journal.pone.0122227 PMC4388645

[275] Lindemann O, Rossaint J, Najder K, Schimmelpfennig S, Hofschröer V, Wälte M, et al. Intravascular adhesion and recruitment of neutrophils in response to CXCL1 depends on their TRPC6 channels. J Mol Med. 2020;98(3):349–60. 31950205 10.1007/s00109-020-01872-4 PMC7080674

[276] Liu Z, Yang J, Zhang X, Xu P, Zhang T, Yang Z. Developmental changes in the expression and function of TRPC6 channels related the F-actin organization during differentiation in podocytes. Cell Calcium. 2015;58(6):541–8. 26363733 10.1016/j.ceca.2015.09.001

[277] Riazanski V, Gabdoulkhakova AG, Boynton LS, Eguchi RR, Deriy LV, Hogarth DK, et al. TRPC6 channel translocation into phagosomal membrane augments phagosomal function. Proc Natl Acad Sci. 2015;112(47):E6486–95. 26604306 10.1073/pnas.1518966112 PMC4664321

[278] Spassova MA, Hewavitharana T, Xu W, Soboloff J, Gill DL. A common mechanism underlies stretch activation and receptor activation of TRPC6 channels. Proc Natl Acad Sci. 2006;103(44):16586–91. 17056714 10.1073/pnas.0606894103 PMC1637625

[279] Serafini N, Dahdah A, Barbet G, Demion M, Attout T, Gautier G, et al. The TRPM4 channel controls monocyte and macrophage, but not neutrophil, function for survival in sepsis. J Immunol. 2012;189(7):3689–99. 22933633 10.4049/jimmunol.1102969

[280] Morita H, Honda A, Inoue R, Ito Y, Abe K, Nelson MT, Brayden JE. Membrane stretch-induced activation of a TRPM4-like nonselective cation channel in cerebral artery myocytes. J Pharmacol Sci. 2007;103(4):417–26. 17420615 10.1254/jphs.fp0061332

[281] Gerzanich V, Kwon MS, Woo SK, Ivanov A, Simard JM. SUR1-TRPM4 channel activation and phasic secretion of MMP-9 induced by tPA in brain endothelial cells. PLoS ONE. 2018;13(4):e0195526. 29617457 10.1371/journal.pone.0195526 PMC5884564

[282] Son M-J, Kim J-C, Kim SW, Chidipi B, Muniyandi J, Singh TD, et al. Shear stress activates monovalent cation channel transient receptor potential melastatin subfamily 4 in rat atrial myocytes via type 2 inositol 1,4,5-trisphosphate receptors and Ca2+release. J Physiol. 2016;594(11):2985–3004. 26751048 10.1113/JP270887 PMC4887694

[283] Zeng Z, Inoue K, Sun H, Leng T, Feng X, Zhu L, Xiong Z-G. TRPM7 regulates vascular endothelial cell adhesion and tube formation. Am J Physiol Cell Physiol. 2015;308(4):C308–18. 25472964 10.1152/ajpcell.00275.2013 PMC4329423

[284] Nauli SM, Kawanabe Y, Kaminski JJ, Pearce WJ, Ingber DE, Zhou J. Endothelial cilia are fluid shear sensors that regulate calcium signaling and nitric oxide production through polycystin-1. Circulation. 2008;117(9):1161–71. 18285569 10.1161/CIRCULATIONAHA.107.710111 PMC3071982

[285] Majhi RK, Sahoo SS, Yadav M, Pratheek BM, Chattopadhyay S, Goswami C. Functional expression of TRPV channels in T cells and their implications in immune regulation. FEBS J. 2015;282(14):2661–81. 25903376 10.1111/febs.13306

[286] Luo H, Saubamea B, Chasseigneaux S, Cochois V, Smirnova M, Glacial F, et al. Molecular and functional study of transient receptor potential vanilloid 1–4 at the rat and human blood-brain barrier reveals interspecies differences. Front Cell Dev Biol. 2020. 10.3389/fcell.2020.578514. 33262985 10.3389/fcell.2020.578514 PMC7686441

[287] Bertin S, Aoki-Nonaka Y, De Jong PR, Nohara LL, Xu H, Stanwood SR, et al. The ion channel TRPV1 regulates the activation and proinflammatory properties of CD4+ T cells. Nat Immunol. 2014;15(11):1055–63. 25282159 10.1038/ni.3009 PMC4843825

[288] Heiner I, Eisfeld J, Halaszovich CR, Wehage E, Jüngling E, Zitt C, Lückhoff A. Expression profile of the transient receptor potential (TRP) family in neutrophil granulocytes: evidence for currents through long TRP channel 2 induced by ADP-ribose and NAD. Biochem J. 2003;371(3):1045–53. 12564954 10.1042/BJ20021975 PMC1223343

[289] Pottosin I, Delgado-Enciso I, Bonales-Alatorre E, Nieto-Pescador MG, Moreno-Galindo EG, Dobrovinskaya O. Mechanosensitive Ca2+-permeable channels in human leukemic cells: pharmacological and molecular evidence for TRPV2. Biochim Biophys Acta (BBA) - Biomembranes. 2015;1848(1):51–9. 25268680 10.1016/j.bbamem.2014.09.008

[290] Nagasawa M, Kojima I. Translocation of TRPV2 channel induced by focal administration of mechanical stress. Physiol Rep. 2015. 10.1481/phy2.12296. 25677550 10.14814/phy2.12296 PMC4393204

[291] Marrelli SP, O’Neil RG, Brown RC, Bryan RM. PLA2 and TRPV4 channels regulate endothelial calcium in cerebral arteries. Am J Physiol-Heart Circ Physiol. 2007;292(3):H1390–7. 17071727 10.1152/ajpheart.01006.2006

[292] Matthews BD, Thodeti CK, Tytell JD, Mammoto A, Overby DR, Ingber DE. Ultra-rapid activation of TRPV4 ion channels by mechanical forces applied to cell surface β1 integrins. Integr Biol. 2010;2(9):435. 10.1039/c0ib00034e PMC3147167 20725677

[293] Vriens J, Watanabe H, Janssens A, Droogmans G, Voets T, Nilius B. Cell swelling, heat, and chemical agonists use distinct pathways for the activation of the cation channel TRPV4. Proc Natl Acad Sci. 2004;101(1):396–401. 14691263 10.1073/pnas.0303329101 PMC314196

[294] Yin J, Michalick L, Tang C, Tabuchi A, Goldenberg N, Dan Q, et al. Role of transient receptor potential vanilloid 4 in neutrophil activation and acute lung injury. Am J Respir Cell Mol Biol. 2016;54(3):370–83. 26222277 10.1165/rcmb.2014-0225OC

[295] Hamanaka K, Jian M-Y, Townsley MI, King JA, Liedtke W, Weber DS, et al. TRPV4 channels augment macrophage activation and ventilator-induced lung injury. Am J Physiol-Lung Cell Mol Physiol. 2010;299(3):L353–62. 20562229 10.1152/ajplung.00315.2009 PMC2951075

[296] Vassilieva IO, Negulyaev YA, Marakhova II, Semenova SB. TRPV5 and TRPV6 calcium channels in human T cells. Cell Tissue Biol. 2008;2(6):584–9. 19140341

[297] Vassilieva IO, Tomilin VN, Marakhova II, Shatrova AN, Negulyaev YA, Semenova SB. Expression of transient receptor potential vanilloid channels TRPV5 and TRPV6 in human blood lymphocytes and jurkat leukemia T cells. J Membr Biol. 2013;246(2):131–40. 23111462 10.1007/s00232-012-9511-x

[298] Michalick L, Kuebler WM. TRPV4—a missing link between mechanosensation and immunity. Front Immunol. 2020. 10.3389/fimmu.2020.00413/full. 32210976 10.3389/fimmu.2020.00413 PMC7076180

[299] Hartmannsgruber V, Heyken W-T, Kacik M, Kaistha A, Grgic I, Harteneck C, et al. Arterial response to shear stress critically depends on endothelial TRPV4 expression. PLoS ONE. 2007;2(9):e827. 17786199 10.1371/journal.pone.0000827 PMC1959246

[300] Baratchi S, Knoerzer M, Khoshmanesh K, Mitchell A, McIntyre P. Shear stress regulates TRPV4 channel clustering and translocation from adherens junctions to the basal membrane. Sci Rep. 2017;7(1):15942. 29162902 10.1038/s41598-017-16276-7 PMC5698423

[301] Swain SM, Liddle RA. Shear stress-induced pathological changes in endothelial cells occur through Piezo1 activation of TRPV4. 2020. 10.1074/jbc.RA120.015059 PMC7948745 33298523

[302] Mendoza SA, Fang J, Gutterman DD, Wilcox DA, Bubolz AH, Li R, et al. TRPV4-mediated endothelial Ca2+ influx and vasodilation in response to shear stress. Am J Physiol Heart Circ Physiol. 2010;298(2):H466–76. 19966050 10.1152/ajpheart.00854.2009 PMC2822567

[303] Rajasekhar P, Poole DP, Veldhuis NA. Role of nonneuronal TRPV4 signaling in inflammatory processes. Adv Pharmacol. 2017;79:117–39. 28528666 10.1016/bs.apha.2017.03.002

[304] Thodeti CK, Matthews B, Ravi A, Mammoto A, Ghosh K, Bracha AL, Ingber DE. TRPV4 channels mediate cyclic strain-induced endothelial cell reorientation through integrin-to-integrin signaling. Circ Res. 2009;104(9):1123–30. 19359599 10.1161/CIRCRESAHA.108.192930 PMC2754067

[305] Song X, Sun Z, Chen G, Shang P, You G, Zhao J, et al. Matrix stiffening induces endothelial dysfunction via the TRPV4/microRNA-6740/endothelin-1 mechanotransduction pathway. Acta Biomater. 2019;100:52–60. 31606530 10.1016/j.actbio.2019.10.013

[306] Dutta B, Goswami R, Rahaman SO. TRPV4 plays a role in matrix stiffness-induced macrophage polarization. Front Immunol. 2020;11:570195. 33381111 10.3389/fimmu.2020.570195 PMC7767862

[307] Inoue R, Jian Z, Kawarabayashi Y. Mechanosensitive TRP channels in cardiovascular pathophysiology. Pharmacol Ther. 2009;123(3):371–85. 19501617 10.1016/j.pharmthera.2009.05.009

[308] Narita K, Sasamoto S, Koizumi S, Okazaki S, Nakamura H, Inoue T, Takeda S. TRPV4 regulates the integrity of the blood-cerebrospinal fluid barrier and modulates transepithelial protein transport. FASEB J. 2015;29(6):2247–59. 25681460 10.1096/fj.14-261396

[309] Rosenkranz SC, Shaposhnykov A, Schnapauff O, Epping L, Vieira V, Heidermann K, et al. TRPV4-mediated regulation of the blood brain barrier is abolished during inflammation. Front Cell Dev Biol. 2020;8:849. 32974355 10.3389/fcell.2020.00849 PMC7481434

[310] Potla R, Hirano-Kobayashi M, Wu H, Chen H, Mammoto A, Matthews BD, Ingber DE. Molecular mapping of transmembrane mechanotransduction through the β1 integrin-CD98hc-TRPV4 axis. J Cell Sci. 2020. 10.1242/jcs.248823. 32989042 10.1242/jcs.248823 PMC7657480

[311] Strotmann R, Schultz G, Plant TD. Ca2+-dependent potentiation of the nonselective cation channel TRPV4 is mediated by a c-terminal calmodulin binding site. J Biol Chem. 2003;278(29):26541–9. 12724311 10.1074/jbc.M302590200

[312] Zhao H, Zhang K, Tang R, Meng H, Zou Y, Wu P, et al. TRPV4 blockade preserves the blood-brain barrier by inhibiting stress fiber formation in a rat model of intracerebral hemorrhage. Front Mol Neurosci. 2018. 10.3389/fnmol.2018.00097. 29636662 10.3389/fnmol.2018.00097 PMC5880899

[313] Fabian A, Bertrand J, Lindemann O, Pap T, Schwab A. Transient receptor potential canonical channel 1 impacts on mechanosignaling during cell migration. Pflügers Arch Eur J Physiol. 2012;464(6):623–30. 23053481 10.1007/s00424-012-1169-9

[314] Eijkelkamp N, Quick K, Wood JN. Transient receptor potential channels and mechanosensation. Annu Rev Neurosci. 2013;36:519–46. 23750514 10.1146/annurev-neuro-062012-170412

[315] Yao X, Garland CJ. Recent developments in vascular endothelial cell transient receptor potential channels. Circ Res. 2005;97(9):853–63. 16254217 10.1161/01.RES.0000187473.85419.3e

[316] Hicks K, O’Neil RG, Dubinsky WS, Brown RC. TRPC-mediated actin-myosin contraction is critical for BBB disruption following hypoxic stress. Am J Physiol Cell Physiol. 2010;298(6):C1583–93. 20164382 10.1152/ajpcell.00458.2009 PMC2889642

[317] Trebak M, Lemonnier L, Smyth JT, Vazquez G, Putney JW Jr. Phospholipase C-coupled receptors and activation of TRPC channels. Handb Exp Pharmacol. 2007;179:593–614. 10.1007/978-3-540-34891-7_35 17217081

[318] Ningoo M, Plant LD, Greka A, Logothetis DE. PIP(2) regulation of TRPC5 channel activation and desensitization. J Biol Chem. 2021;296:100726. 33933453 10.1016/j.jbc.2021.100726 PMC8191310

[319] Adebiyi A, Thomas-Gatewood CM, Leo MD, Kidd MW, Neeb ZP, Jaggar JH. An elevation in physical coupling of type 1 inositol 1,4,5-trisphosphate (IP 3) receptors to transient receptor potential 3 (TRPC3) channels constricts mesenteric arteries in genetic hypertension. Hypertension. 2012;60(5):1213–9. 23045459 10.1161/HYPERTENSIONAHA.112.198820 PMC3632264

[320] Freichel M, Vennekens R, Olausson J, Stolz S, Philipp SE, Weissgerber P, Flockerzi V. Functional role of TRPC proteins in native systems: implications from knockout and knock-down studies. J Physiol. 2005;567(Pt 1):59–66. 15975974 10.1113/jphysiol.2005.092999 PMC1474153

[321] Singh I, Knezevic N, Ahmmed GU, Kini V, Malik AB, Mehta D. Gαq-TRPC6-mediated Ca2+ entry induces RhoA activation and resultant endothelial cell shape change in response to thrombin. J Biol Chem. 2007;282(11):7833–43. 17197445 10.1074/jbc.M608288200

[322] Thilo F, Vorderwülbecke BJ, Marki A, Krueger K, Liu Y, Baumunk D, et al. Pulsatile atheroprone shear stress affects the expression of transient receptor potential channels in human endothelial cells. Hypertension. 2012;59(6):1232–40. 22566504 10.1161/HYPERTENSIONAHA.111.183608

[323] Du J, Fu J, Xia XM, Shen B. The functions of TRPP2 in the vascular system. Acta Pharmacol Sin. 2016;37(1):13–8. 26725733 10.1038/aps.2015.126 PMC4722977

[324] Qu Y-Y, Wang L-M, Zhong H, Liu Y-M, Tang N, Zhu L-P, et al. TRPC1 stimulates calcium-sensing receptor-induced store-operated Ca2+ entry and nitric oxide production in endothelial cells. Mol Med Rep. 2017;16(4):4613–9. 28791397 10.3892/mmr.2017.7164 PMC5647016

[325] Sharif-Naeini R, Folgering JH, Bichet D, Duprat F, Lauritzen I, Arhatte M, et al. Polycystin-1 and -2 dosage regulates pressure sensing. Cell. 2009;139(3):587–96. 19879844 10.1016/j.cell.2009.08.045

[326] Aboualaiwi WA, Takahashi M, Mell BR, Jones TJ, Ratnam S, Kolb RJ, Nauli SM. Ciliary polycystin-2 is a mechanosensitive calcium channel involved in nitric oxide signaling cascades. Circ Res. 2009;104(7):860–9. 19265036 10.1161/CIRCRESAHA.108.192765 PMC3085025

[327] Cha S-K, Kim J-H, Huang C-L. Flow-induced activation of TRPV5 and TRPV6 channels stimulates Ca(2+)-activated K(+) channel causing membrane hyperpolarization. Biochem Biophys Acta. 2013;1833(12):3046–53. 24001793 10.1016/j.bbamcr.2013.08.017 PMC3932323

[328] Shuvaev A, Kuvacheva NV, Morgun A, Khilazheva E, Salmina AB. The role of ion channels expressed in cerebral endothelial cells in the functional integrity of the blood-brain barrier (review). Sovremennye tehnologii v medicine. 2016;8:241–50.

[329] Yankaskas CL, Bera K, Stoletov K, Serra SA, Carrillo-Garcia J, Tuntithavornwat S, et al. The fluid shear stress sensor TRPM7 regulates tumor cell intravasation. Sci Adv. 2021;7(28):3457. 10.1126/sciadv.abh3457 PMC8270498 34244134

[330] Numata T, Shimizu T, Okada Y. Direct mechano-stress sensitivity of TRPM7 channel. Cell Physiol Biochem. 2007;19(1–4):1–8. 17310095 10.1159/000099187

[331] Liu Y-S, Liu Y-A, Huang C-J, Yen M-H, Tseng C-T, Chien S, Lee OK. Mechanosensitive TRPM7 mediates shear stress and modulates osteogenic differentiation of mesenchymal stromal cells through osterix pathway. Sci Rep. 2015;5(1):16522. 26558702 10.1038/srep16522 PMC4642269

[332] Alvarado MG, Thakore P, Earley S. Transient receptor potential channel ankyrin 1: a unique regulator of vascular function. Cells. 2021. 10.3390/cells10051167. 34064835 10.3390/cells10051167 PMC8151290

[333] Moparthi L, Zygmunt PM. Human TRPA1 is an inherently mechanosensitive bilayer-gated ion channel. Cell Calcium. 2020;91:102255. 32717533 10.1016/j.ceca.2020.102255

[334] Rostami A, Ciric B. Role of Th17 cells in the pathogenesis of CNS inflammatory demyelination. J Neurol Sci. 2013;333(1–2):76–87. 23578791 10.1016/j.jns.2013.03.002 PMC3726569

[335] Rudolph H, Klopstein A, Gruber I, Blatti C, Lyck R, Engelhardt B. Postarrest stalling rather than crawling favors CD8 + over CD4 + T-cell migration across the blood–brain barrier under flow in vitro. Eur J Immunol. 2016;46(9):2187–203. 27338806 10.1002/eji.201546251 PMC5113696

[336] Abadier M, Pramod AB, McArdle S, Marki A, Fan Z, Gutierrez E, et al. Effector and regulatory T cells roll at high shear stress by inducible tether and sling formation. Cell Rep. 2017;21(13):3885–99. 29281835 10.1016/j.celrep.2017.11.099 PMC5786164

[337] Sundd P, Gutierrez E, Koltsova EK, Kuwano Y, Fukuda S, Pospieszalska MK, et al. ‘Slings’ enable neutrophil rolling at high shear. Nature. 2012;488(7411):399–403. 22763437 10.1038/nature11248 PMC3433404

[338] Dusi S, Angiari S, Pietronigro EC, Lopez N, Angelini G, Zenaro E, et al. LFA-1 controls Th1 and Th17 motility behavior in the inflamed central nervous system. Front Immunol. 2019;10:2436. 31681316 10.3389/fimmu.2019.02436 PMC6813462

[339] Woodfin A, Voisin M-B, Nourshargh S. PECAM-1: a multi-functional molecule in inflammation and vascular biology. Arterioscler Thromb Vasc Biol. 2007;27(12):2514–23. 17872453 10.1161/ATVBAHA.107.151456

[340] Kalinowska A, Losy J. PECAM-1, a key player in neuroinflammation. Eur J Neurol. 2006;13(12):1284–90. 17116209 10.1111/j.1468-1331.2006.01640.x

[341] Chien S. Effects of disturbed flow on endothelial cells. Ann Biomed Eng. 2008;36(4):554–62. 18172767 10.1007/s10439-007-9426-3 PMC3718045

[342] Alexander JS, Alexander BC, Eppihimer LA, Goodyear N, Haque R, Davis CP, et al. Inflammatory mediators induce sequestration of VE-cadherin in cultured human endothelial cells. Inflammation. 2000;24(2):99–113. 10718113 10.1023/a:1007025325451

[343] Koivisto A-P, Belvisi MG, Gaudet R, Szallasi A. Advances in TRP channel drug discovery: from target validation to clinical studies. Nature Reviews Drug Discovery. 2021. 10.1038/s41573-021-00268-4 PMC8442523 34526696

[344] Miller F, Björnsson M, Svensson O, Karlsten R. Experiences with an adaptive design for a dose-finding study in patients with osteoarthritis. Contemp Clin Trials. 2014;37(2):189–99. 24394343 10.1016/j.cct.2013.12.007

